# Opening of glutamate receptor channel to subconductance levels

**DOI:** 10.1038/s41586-022-04637-w

**Published:** 2022-04-20

**Authors:** Maria V. Yelshanskaya, Dhilon S. Patel, Christopher M. Kottke, Maria G. Kurnikova, Alexander I. Sobolevsky

**Affiliations:** 1 Department of Biochemistry and Molecular Biophysics, Columbia University, New York, NY USA; 2 Chemistry Department, Carnegie Mellon University, Pittsburgh, PA USA

**Keywords:** Cryoelectron microscopy, Permeation and transport

## Abstract

Ionotropic glutamate receptors (iGluRs) are tetrameric ligand-gated ion channels that open their pores in response to binding of the agonist glutamate^1–3^. An ionic current through a single iGluR channel shows up to four discrete conductance levels (O1–O4)^4–6^. Higher conductance levels have been associated with an increased number of agonist molecules bound to four individual ligand-binding domains (LBDs)^6–10^. Here we determine structures of a synaptic complex of AMPA-subtype iGluR and the auxiliary subunit γ2 in non-desensitizing conditions with various occupancy of the LBDs by glutamate. We show that glutamate binds to LBDs of subunits B and D only after it is already bound to at least the same number of LBDs that belong to subunits A and C. Our structures combined with single-channel recordings, molecular dynamics simulations and machine-learning analysis suggest that channel opening requires agonist binding to at least two LBDs. Conversely, agonist binding to all four LBDs does not guarantee maximal channel conductance and favours subconductance states O1 and O2, with O3 and O4 being rare and not captured structurally. The lack of subunit independence and low efficiency coupling of glutamate binding to channel opening underlie the gating of synaptic complexes to submaximal conductance levels, which provide a potential for upregulation of synaptic activity.

## Abstract (web summary)

Cryo-EM structures of AMPA receptor with the subunit γ2 in non-desensitizing conditions at low glutamate concentrations disprove the one-to-one link between the number of glutamate-bound subunits and ionotropic glutamate receptor conductance.

## Main

iGluRs are tetrameric ion channels that mediate the majority of excitatory neurotransmission in the central nervous system^1^. iGluRs share a common architecture that consists of the following layers: an upper extracellular layer of amino-terminal domains (ATDs), which are involved in receptor assembly and regulation; a layer of transmembrane domains (TMDs), which form an ion-conducting channel; a layer of LBDs sandwiched between ATD and TMD layers; and a layer of intracellular domains (ICDs), which have not been structurally resolved^3^. In the ATD and LBD layers, the domains have a dimer-of-dimers arrangement. Each LBD comprises two polypeptide stretches (S1 and S2) that assemble into a clamshell-like structure with an agonist-binding site between the upper (D1) and lower (D2) lobes of the clamshell. Agonist binding results in closure of the LBD clamshell^11^ and initiates the process of gating that culminates in ion conductance through the channel^2^.

Three iGluR subtypes—AMPA, kainate and NMDA receptors—are activated by the agonist glutamate (Glu). Activation of these iGluRs is characterized by single-channel currents that appear from the baseline level (C) in a stepwise manner and reach up to four (sub)conductance levels (O1–O4) when recorded in neuronal preparations^4–6,12–16^. AMPA receptors are the fastest iGluRs, and up to four (sub)conductance levels have been observed for heterologously expressed receptors composed of each type of GluA1–GluA4 subunit, their combinations or their complexes with auxiliary subunits^7–10,17–29^. Similarly, kainate receptors show multiple conductance levels when expressed alone or in the presence of Neto auxiliary subunits^22,26,30,31^. It has become generally accepted that multiple conductance levels arise from individual iGluR subunits that independently gate the channel, with the average conductance determined by how many subunits are bound to an agonist^6–10,15,23,24,32,33^. However, deviations from independence of subunits and subunit cooperativity have been reported for NMDA^34^ and kainate^6^ receptors, as well as AMPA receptors at low Glu concentrations and negative voltages^7^ or in the presence of noncompetitive inhibitors^23^.

Although numerous functional studies support a direct link between ion channel conductance and several independent or nearly independent subunits bound to agonists, this view lacks structural support. Indeed, the only available structures of conducting iGluRs are open-state structures of AMPA receptors with all four LBDs bound to agonists^35–37^. iGluR structures with only a fraction of subunits bound to agonist have not been reported so far. To fill this gap in knowledge, we solved structures of AMPA receptor complexes with an auxiliary subunit γ2 or stargazin^38^ in non-desensitizing conditions at low Glu concentrations, thus favouring incomplete occupancy of the LBD by an agonist. Contrary to the common view on AMPA receptor activation, we found strong cooperativity and allosteric interactions between receptor subunits. A minimum of two subunits bound to an agonist are required to open the GluA2–γ2 complex to the lowest conducting level O1, yet two bound agonists is also sufficient to reach the higher conductance levels O2 and O3. At the same time, Glu binding to all four GluA2 LBD subunits does not necessarily result in maximal ion channel conductance. In fact, the channel can reside in O1 or O2 with all four LBDs bound to Glu and their clamshells closed. These results disprove the one-to-one link between the number of Glu-bound subunits and iGluR conductance and suggest a more complex relationship between agonist binding and channel opening than previously thought.

## Functional characterization

**Fig. 1:**
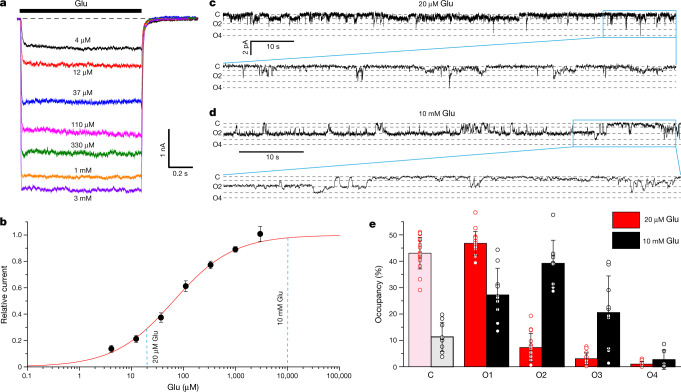
Multilevel conductance at low and high Glu concentrations. **a**, Superposition of typical whole-cell currents recorded at –60 mV membrane potential from a HEK-293T cell expressing GluA2–γ2 in response to 1-s applications of Glu at different concentrations in the continuous presence of 100 µM CTZ. **b**, Dose–response curve for Glu measured for the amplitude of currents illustrated in **a** and normalized to their maximal value. The red line illustrates a logistic equation fit with the half maximal effective concentration value of 65 ± 5 µM (*n* = 7). Data are presented as the mean ± s.e.m. **c**, **d**, Representative single-channel currents recorded at –60 mV membrane potential from GluA2–γ2 reconstituted into lipid bilayers in the presence of 100 µM CTZ and 20 µM (**c**) or 10 mM (**d**) Glu. Horizontal dashed lines indicate different conductance levels. The conductance level of the closed channel is labelled (**c**). **e**, Relative occupancy of conductance levels at 20 µM and 10 mM of Glu averaged over *n* = 14 (*n* = 12 for O4 as O4 was not observed in some experiments) and *n* = 10 (*n* = 7 for O4) independent experiments illustrated in **c** and **d**, respectively, with the mean conductance of 8.2 pS for O1, 18.8 pS for O2, 27.0 pS for O3 and 37.0 pS for O4. Data are presented as the mean ± s.d.

The auxiliary subunit γ2 promotes opening of AMPA receptor channels^27,39–41^. To study agonist-dependent activation, we fused the N terminus of γ2 to the carboxy terminus of the AMPA receptor subunit GluA2 (modified calcium-permeable rat GluA2_flip_ subunit with Q586 at the Q/R site; Methods). In the presence of the positive allosteric modulator cyclothiazide (CTZ), Glu-induced whole-cell GluA2–γ2-mediated currents did not show apparent desensitization (Fig. 1a). GluA2–γ2 activation was concentration-dependent (Fig. 1b), with a half-maximal effective concentration value of 65 ± 5 µM (*n* = 7, mean ± s.e.m.). To examine the activation of individual receptors, we reconstituted purified GluA2–γ2 into lipid bilayers and recorded single-channel currents at low (20 µM) and high (10 mM) Glu concentrations in the continuous presence of 100 µM CTZ to block desensitization (Fig. 1c, d). At both Glu concentrations, GluA2–γ2-mediated single-channel currents showed four conductance levels (O1–O4). O1 was the predominant conductance level at the low Glu concentration, whereas O2 dominated at the high Glu concentration (Fig. 1e), which is consistent with previous observations^18,28,42^.

## Structural ensemble

**Fig. 2:**
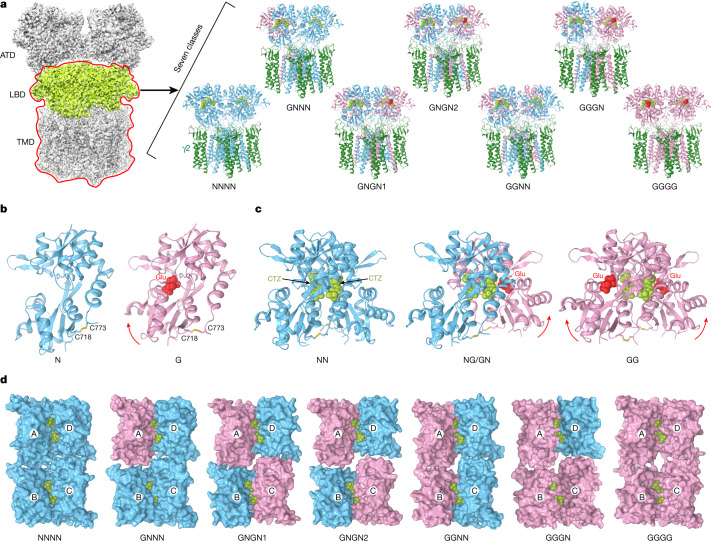
Structural ensemble at low Glu concentration. **a**, Particles of GluA2–γ2 collected at 20 µM Glu and 100 µM CTZ produced an average three-dimensional reconstruction (left) and classified on the basis of the LBD layer (light green) and focused on the LBD–TMD (red contour) into seven structures (right). GluA2 subunits not bound to Glu (N) are shown in blue, Glu-bound (G) in pink and γ2 in dark green. **b**, **c**, Side views of monomers (**b**) and dimers (**c**) of LBD that represent the GluA2–γ2 structural ensemble at low Glu concentration. Glu molecules are shown in ball-and-stick representation (yellow), whereas CTZ (green) and disulfide-linked cysteines C718 and C773 are shown as sticks. LBD clamshell closure in response to Glu binding is indicated by red arrows. **d**, Top views of LBD tetramers that represent the GluA2–γ2 structural ensemble are shown in surface representation and viewed from the ion channel side.

**Extended Data Fig. 1:**
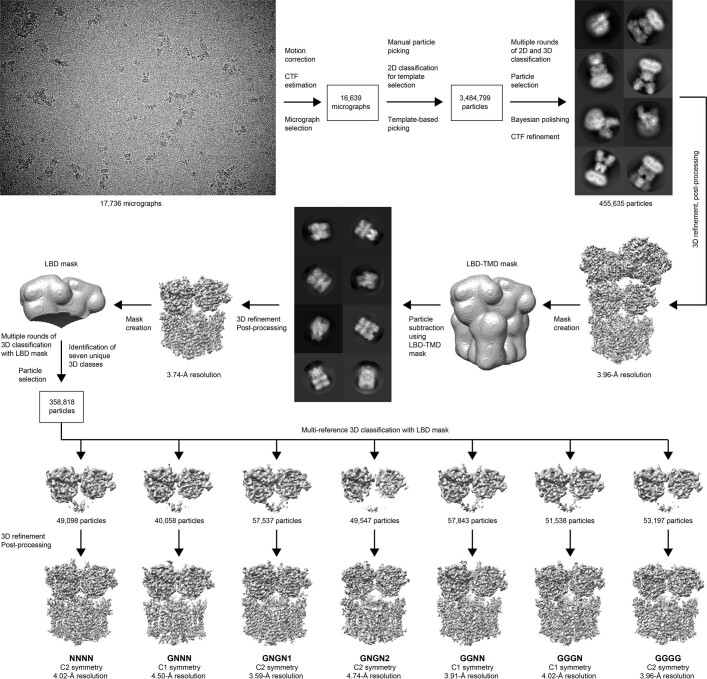
3D reconstruction workflow .

**Extended Data Fig. 2:**
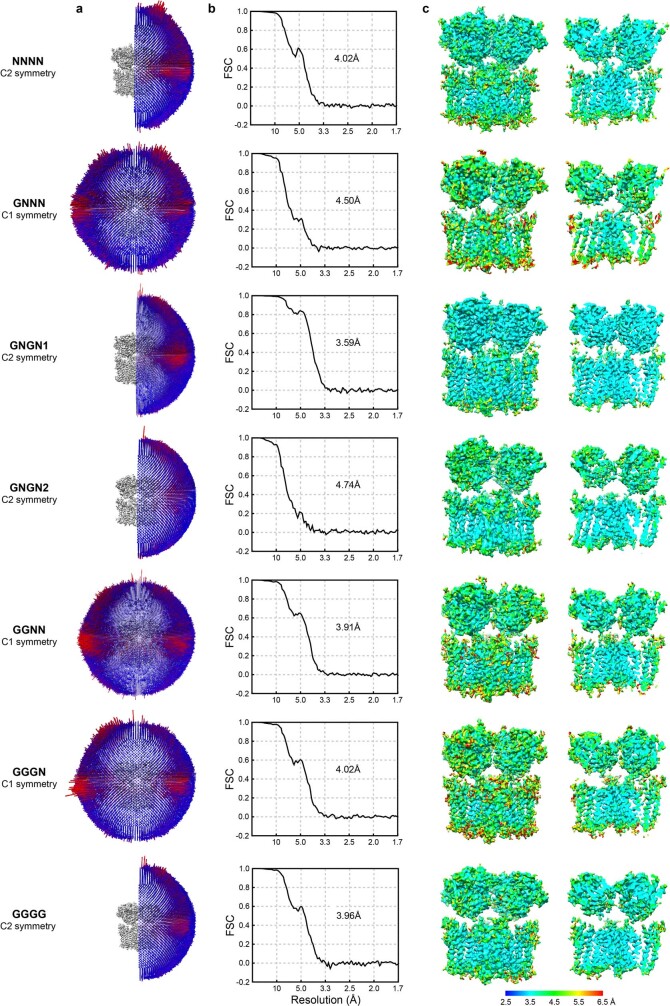
Characteristics of cryo-EM reconstructions. **a**, Euler angle distribution of particles contributing to the final reconstructions with larger red cylinders representing orientations comprising more particles. **b**, FSC curves calculated between half maps, with the resolution range estimated using the FSC = 0.143 criterion. **c**, Local resolution presented as coloring of the cryo-EM maps in UCSF Chimera 1.14.

**Extended Data Fig. 3:**
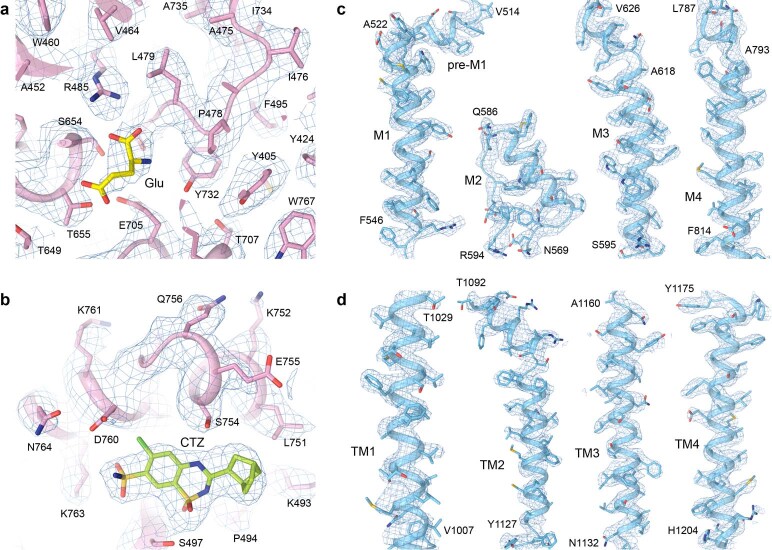
Cryo-EM density. Fragments of cryo-EM density (blue mesh) for GNGN1 structure, focusing on the agonist-binding site bound to Glu (yellow, **a**), LBD interface binding site of CTZ (green, **b**), and TMD segments of GluA2 (**c**) and γ2 (**d**).

To study agonist-dependent activation structurally, we subjected purified GluA2–γ2 to cryogenic electron microscopy (cryo-EM) analyses (Extended Data Figs. 1, 2 and Extended Data Table 1). Previously, we determined the GluA2–γ2 structure in the presence of CTZ and high Glu concentration (100 mM; Protein Data Bank (PDB) ID: 5WEO) and found that the receptor resides primarily in a single conformation, with all four LBD clamshells bound to Glu and closed and each LBD dimer interface harbouring two molecules of CTZ^35^. This time, to characterize the ensemble of receptors activated by different numbers of agonist molecules, we prepared cryo-EM samples in the presence of CTZ (100 µM) and a low Glu concentration (20 µM). In these conditions (Fig. 1b), each GluA2–γ2 complex is expected to bind a various number (zero to four) of Glu molecules. Indeed, seven distinct GluA2–γ2 structures were determined on the basis of different LBD layer conformations (Fig. 2a). In all these structures, the D1 lobes of the back-to-back LBD dimers were tightly bound to each other, and each D1–D1 interface had two bound CTZ molecules represented by well-resolved densities in the cryo-EM maps (Extended Data Fig. 3). Data processing (Extended Data Fig. 1) did not reveal structures with a raptured D1–D1 interface, which provides strong support for the absence of desensitized states in the captured structural ensemble^2,36,43^.

**Extended Data Fig. 4:**
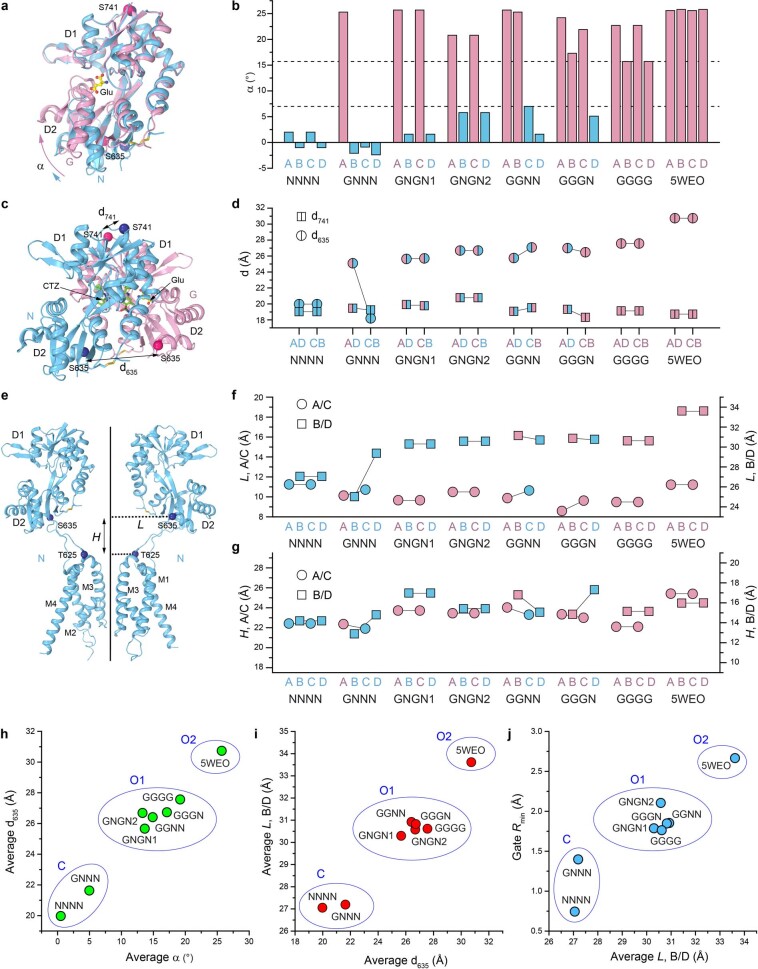
Discrete structural behavior of LBDs. **a**, Upper lobe (D1)-based superposition of the Glu-bound (G, pink) and not bound (N, blue) LBDs, with the relative positioning of the lower lobe (D2) characterized by the clamshell closure angle α and illustrated by arrows. The molecule of Glu is shown as a ball-and-stick model (yellow). Cα atoms of S635 and S741 are shown as spheres. **b**, Clamshell closure angle α measured relative to the apo-state structure (PDB ID: 5L1B) using DynDom, with the values for the Glu-bound (α > 15°, upper dashed line) and not bound (α < 7°, lower dashed line) LBDs shown as pink and blue columns, respectively. **c**, Structure of an NG dimer, with the molecules of CTZ shown in sticks (green). Cross-dimer distances between S635 and S741 are indicated by double arrows and labeled. **d**, Cross-dimer distances for individual dimers in each structure. **e**, LBD-TMD regions for two diagonal subunits in NNNN structure. The distance from S635 to the axis of the overall two-fold symmetry (*L*) and its height over T625 (*H*) are indicated by double arrows and labeled. **f-g**, Values of *L* (**f**) and *H* (**g**) measured for A/C (circles) and B/D (squares) subunits in each structure. **h**, Plot of d635 averaged over AD and CB dimers versus α averaged over all four subunits. Blue ovals highlight the putative assignment of structures to non-conducting C and conducting O1 and O2 states. **i**, Plot of *L* averaged over subunits B and D versus d635 averaged over AD and CB dimers. **j**, Plot of minimum pore radius in the gate versus *L* averaged over subunits B and D.

Differences between the seven structures that represent the structural ensemble at 20 µM Glu are obvious at the levels of individual LBDs, LBD dimers and LBD tetramers. There were two types of individual LBD monomers (Fig. 2b): Glu-bound (G) and not bound (N). Assignment of each individual LBD to the G or N type was unambiguous because of the presence or absence of Glu density in the agonist-binding pocket (Extended Data Fig. 3) and the clearly closed or open LBD clamshell (Fig. 2b), respectively. Indeed, Glu-bound LBDs had clamshell closure angle *α* values larger than 15°, whereas ligand-free clamshells had *α* values smaller than 7° (Extended Data Fig. 4a, b). There were three types of LBD dimers that represented all possible combinations of N and G monomers (Fig. 2c): GG, GN (=NG) and NN. NN and GG dimers had two-fold rotational symmetry, whereas GN dimers were asymmetrical.

Given the equivalence of subunits A and C as well as B and D in iGluR tetramers^3^, the following ten tetrameric arrangements of G and N monomers were possible: NNNN, GNNN, NGNN, GNGN, GGNN, NGGN, NGNG, GGNG, GGGN and GGGG. At 20 µM Glu, the structural ensemble included only six of the possible tetrameric arrangements—NNNN, GNNN, GNGN, GGNN, GGGN and GGGG—with NGNN, NGGN, NGNG and GGNG tetramers not present (Fig. 2d). The GNGN arrangement was presented by two structures (GNGN1 and GNGN2) that had distinct conformations. According to the ensemble composition, Glu can bind to LBDs of subunits B and D only after it is already bound to at least the same number of LBDs that belong to subunits A and C. Such a strict order in Glu binding supports the non-equivalent contribution of subunits to AMPA receptor gating^35,44^.

## Ion channel pore

**Fig. 3:**
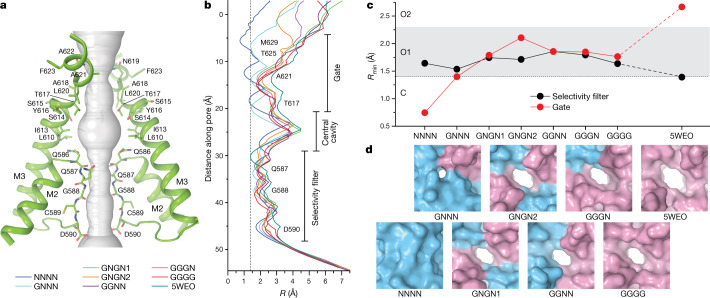
Ion channel pore. **a**, Pore-forming domains in GNGN1 with the residues that line the pore shown as sticks. Only two of four subunits are shown, with the front and back subunits omitted for clarity. The pore profile is shown as a space-filling model (grey). **b**, Pore radius values calculated using HOLE. The vertical dashed line denotes the radius of a water molecule (1.4 Å). **c**, Minimum pore radius values in the selectivity filter (black) and gate region (red). **d**, The gate region of the pore of the indicated structures in surface representation viewed perpendicular to the membrane.

To find out whether the conformational diversity observed at the LBD layer (Fig. 2b–d) translates to the ion channel pore, which includes two narrow regions, the gate and the selectivity filter, we estimated pore radii for all structures of the ensemble (Fig. 3a, b). The channel pore selectivity filter is formed by the extended portions of the re-entrant M2 loop. Five amino acids of the selectivity filter, Q586, Q587, G588, C589 and D590, contribute their backbone carbonyls and polar side chains to make the pore surface electronegative^45^. As the selectivity filter pore radius in all structures was slightly larger than the radius of a water molecule (Fig. 3b), their selectivity filters are expected to permeate both ions and water. Indeed, molecular dynamics (MD) simulations of the transmembrane domain of the structure obtained at high Glu concentration (PDB ID: 5WEO) demonstrated permeation of Na^+^ and K^+^ ions through the selectivity filter^46^. As the selectivity filter in all structures at 20 µM Glu was the same or slightly wider than in the 5WEO structure (Fig. 3c), this region of the pore appears to determine ion selectivity and permeation rather than channel gating.

In contrast to the selectivity filter, the gate region of the pore is formed by mostly hydrophobic residues of the M3 segments, which can prevent the conductance of ions and water through the mechanism of hydrophobic seal^47–50^. Side chains of T617, A621, T625 and M629 contributed to the narrow constriction of the pore at the gate region and determined differences in the pore opening between the structures (Fig. 3a, b). Estimates of the minimal pore radius, *R*_min_, suggested that the gate region is non-conducting in the NNNN and GNNN structures, whereas it can be permeable to water and ions in the GNGN1, GNGN2, GGNN, GGGN and GGGG structures (Fig. 3c, d). In the two non-conducting structures, all four M3 segments were entirely α-helical, with a slightly (one helical turn) unwound M3 in subunit A of the GNNN structure. By contrast, in the conducting structures, the M3 segments in subunits B and D were bent at the gating hinge alanine A618, similar to the 5WEO structure^35^. Of note, the *R*_min_ values in the gate region of the GNGN1, GNGN2, GGNN, GGGN and GGGG structures were similar, being slightly larger in GNGN2 and smaller than in the 5WEO structure obtained at a high Glu concentration. When comparing the *R*_min_ values at the gate region (Fig. 3c) to the pattern of a single-channel activity at low and high Glu concentrations (Fig. 1e), it is tempting to conclude that the NNNN and GNNN structures represent the non-conducting state C (*R*_min_ ≤ 1.4 Å), whereas the GNGN1, GNGN2, GGNN, GGGN and GGGG structures represent the first conductance level O1 (1.4 Å < *R*_min_ ≤ 2.3 Å) and the 5WEO structures represents the second conductance level O2 (*R*_min_ > 2.3 Å).

## Molecular dynamics simulations

**Extended Data Fig. 5:**
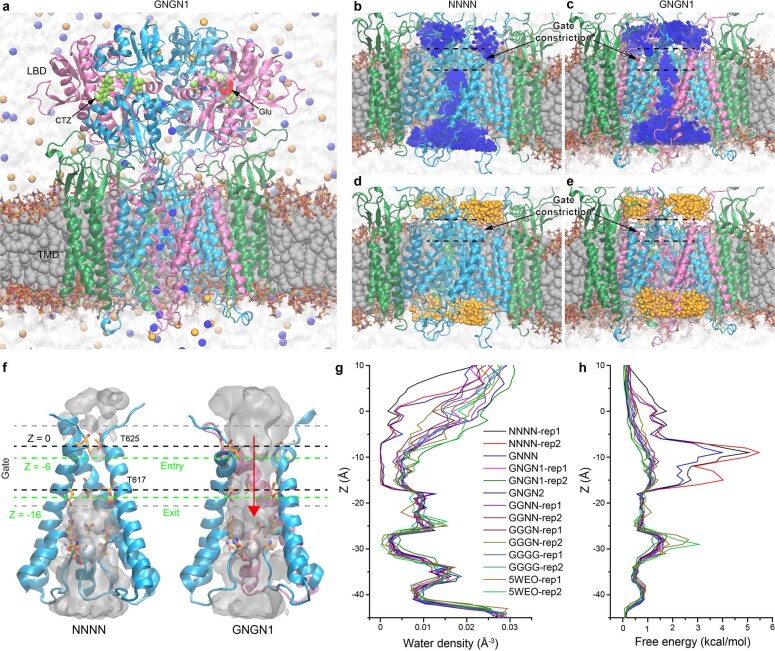
MD simulation system, ion conductance and water density. **a**, A snapshot of the simulated GNGN1 system with AMPA receptor and γ2 subunits shown as blue, pink and green ribbons, lipid bilayer in grey space-filling representation with hydrophilic head groups as sticks, CTZ in pale green, glutamate in red, water as light grey continuum, and Na^+^ and Cl^−^ ions as blue and orange spheres, respectively. **b-e**, MD simulation system for NNNN (**b,d**) and GNGN1 (**c,e**) with integral occupancy of the pore by Na^+^ (**b-c**) and Cl^−^ (**d-e**) ions. **f**, Channel pore in NNNN (closed) and GNGN1 (open) structures with the integral occupancy by water shown as light grey continuum. Water permeation was calculated as the number of water molecules crossing the space between levels Z = –6 Å and Z = –16 Å (dashed green lines) per ns (red arrow). **g**, Water density profiles are shown for all simulated systems. The center of mass of four T625 residues is taken as the origin. **h**, Free energy for resident water to enter the channel. Continuous water occupancy, an indication of water conductance, is observed in all structures, except closed-state structures NNNN and GNNN, which remain non-conducting during microsecond-long simulations. Plots in **g** and **h** are aligned with the structure in (**f**).

To evaluate the structural diversity and stability of the structures at near-physiological conditions, as well as to evaluate our assignment of structures to the conductance levels, we performed various equilibrium and non-equilibrium MD simulations in the presence and absence of an applied voltage. For each cryo-EM structure, we built a model system of the GluA2–γ2 complex embedded in a lipid bilayer and surrounded by water and ions (Extended Data Table 2 and Extended Data Fig. 5a). Equilibrium MD simulations were carried out at room temperature for 1–2 µs (Extended Data Table 2). Open channels exhibited water and permeating ions travelling freely through their gate (Supplementary Videos 1, 2). A continuous integral occupancy of the pore by ions or water over the simulation time course was indicative of an open pore (Extended Data Fig. 5b–h). A continuous integral occupancy of the pore by Na^+^ ions and water were observed in equilibrium simulations of the GNGN1, GNGN2, GGNN, GGGN, GGGG and 5WEO structures, which confirmed that these structures are in the open conducting state (Extended Data Fig. 5c, f–h**)**. By contrast, non-continuous integral occupancy of the pore devoid of Na^+^ ions and water in the gate regions of the NNNN and GNNN structures confirmed that these are non-conducting (Extended Data Fig. 5b, f–h). In simulations of all structures, the negatively charged chloride ions never entered the pore (Extended Data Fig. 5d, e), which provides strong support for the cation selectivity of iGluRs^51^.

## Machine learning analysis

**Fig. 4:**
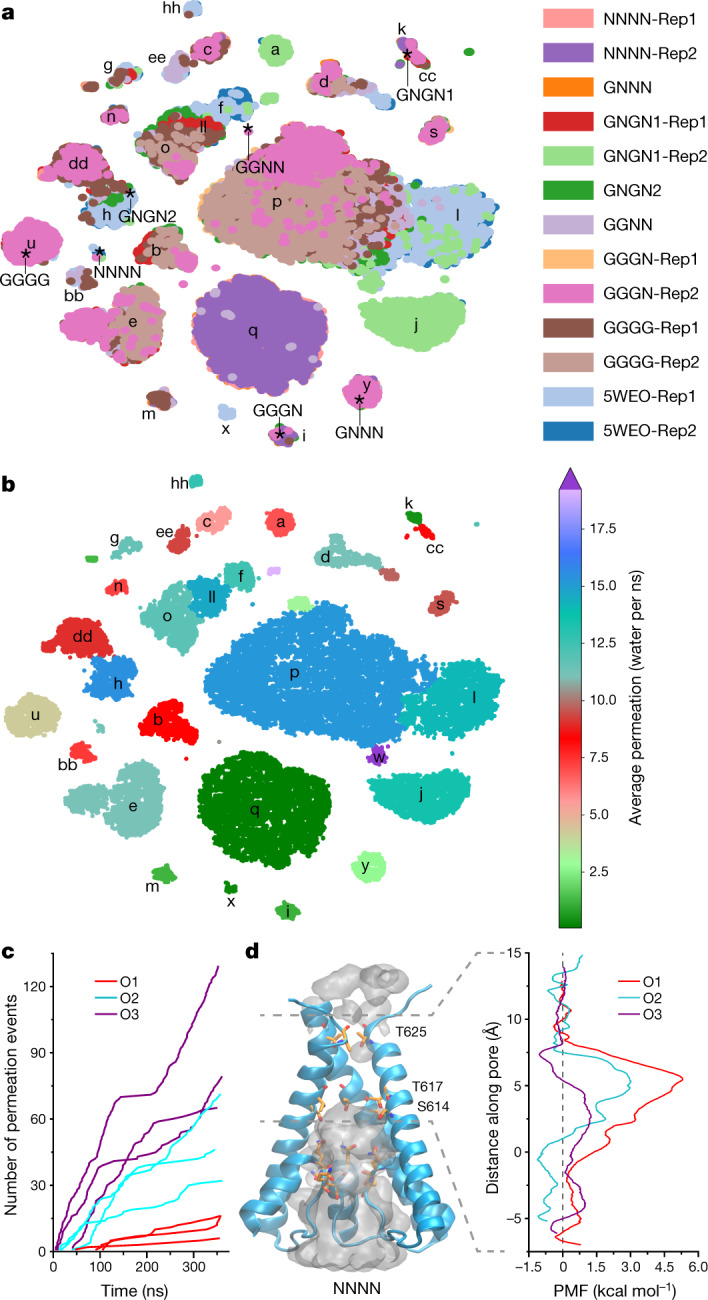
Cluster analysis of structures and permeation. **a**, Clusters identified from *t*-distributed stochastic neighbour embedding (*t*-SNE) clustering of all MD data based on the T617 ${\chi }_{1}$ dihedral angles and pairwise cross-tetramer distances at T625 and T617. Clusters are alphabetically labelled. Positions of cryo-EM structures are indicated with asterisks. Rep, replicate trajectory (Extended Data Table 2). **b**, The same clusters as in **a**, but coloured according to the average water permittivity calculated for points within the cluster. **c**, Cumulative K^+^ ion permeation events during non-equilibrium MD simulations of representative structures for the conducting states O1, O2 and O3 under applied voltage. Each line represents an individual trajectory. **d**, Single K^+^ ion PMF profiles calculated using umbrella sampling simulations for O1, O2 and O3 states. PMF was computed for the ion crossing the gate region as shown (grey dashed lines). The channel pore of the NNNN structure with integral occupancy by water (grey) is shown as a reference.

**Extended Data Fig. 6:**
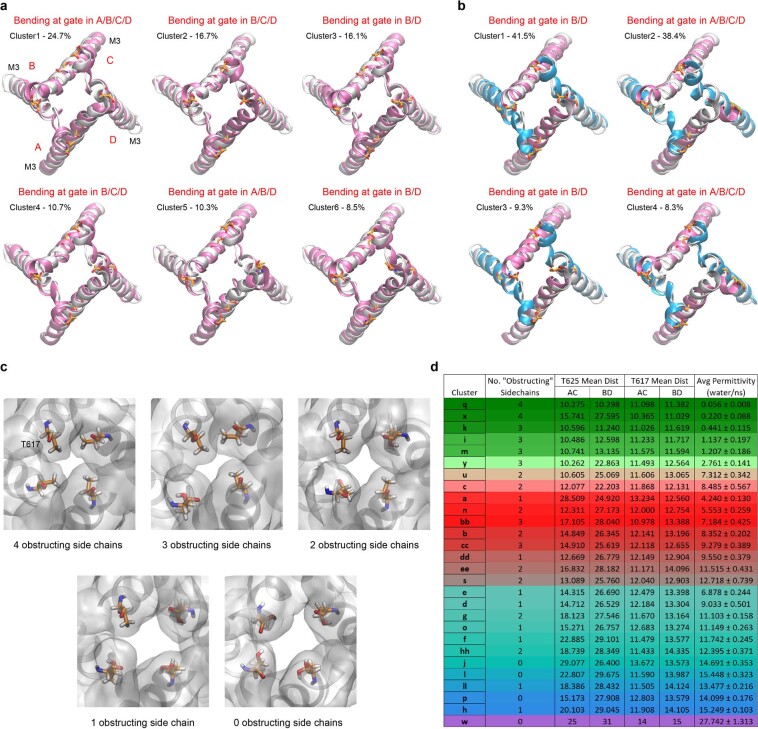
Conformational flexibility of simulated structures and cluster analysis of permeation. **a**, Ensemble of structures (pink) simulated starting with 5WEO structure (grey) and sorted with the k-means clustering algorithm. Structure alignment was done using the backbone atoms C, O, N, Cα, Cβ for residues in the channel gate (S614 to T625) over the total of 16,112 MD frames. Unlike cryo-EM structure, cluster representatives suggest wider opening of the M3 gate with deformation and/or bending in A/C subunits along with bending in B/D subunits during the simulations. Only the first six out of the total of 10 clusters are shown, which represent more than 85% of population. **b**, Representative M3-aligned structures from RMSD clustering (using backbone atoms C, O, N, Cα, Cβ) of the M3 gate residues (S614 to T625) performed over the total of 12,493 frames from both replicas of GNGN1. For the reference, the M3 segments of the cryo-EM structure are shown in grey. The largest cluster, Cluster1, shows similar to the cryo-EM structure bending at B/D. The second largest cluster, Cluster2, which represents ~38% of the population, shows wider opening of the M3 gate, with deformation and/or bending in A/C subunits along with bending in B/D subunits during MD simulations. Only the first four out of five clusters are shown, which represents more than 95% population. **c**, Five conformational configurations of T617 determining permeation of water. **d**, Clusters listed in order of increasing water permeation, computed as a number of water molecules crossing the gate region in one direction per ns. Distances are in Angstroms.

All simulated structures, although stable on average (Supplementary Table 1), exhibited conformational flexibility and diversity (Extended Data Figure 6a, b). We used machine-learning approaches to analyse the entire ensemble of the MD-generated conformations of the M3 gate region described by a large set of geometric features, such as pairwise distances between atoms in the neighbouring subunits and across the tetramer, dihedral angles of the residue backbone and side chain, and an area of the pore at T617, A621 and T625 residues. Machine-learning dimensionality reduction algorithms found a subset of seven geometric features that uniquely described the state of the pore, including the AC and BD inter-subunit distances between T625 Cα atoms (across the pore) and the T617 side chain conformation. All structures were sorted into clusters by similarity in these features (Fig. 4a, Extended Data Fig. 6 and Supplementary Tables 2–5). Importantly, simulations starting from a given cryo-EM structure were sampling several structural clusters in a single trajectory (Fig. 4a and Supplementary Table 5).

**Fig. 5:**
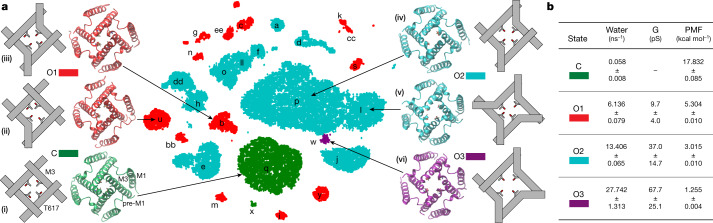
MD-predicted conductance levels. **a**, Centre, *t*-SNE structural clusters are coloured by water permittivity. Periphery, schematics and MD models of gate regions in structures representing different conductance levels, including closed (C) with all four M3 helices straight and four T617 obstructing the pore (i), O1 with a single M3 bent and two T617 obstructing the pore (ii), O1 with two M3 bent and two T617 obstructing the pore (iii), O2 with two M3 bent and no T617 obstructions (iv), O2 with four M3 bent and no T617 obstructions (v) and O3 with three M3 helices bent and no T617 obstructions (vi). **b**, Water permittivity, ion conductance (G) and PMFs for C, O1, O2 and O3 obtained from MD simulations of representative structures. Data are the mean ± s.d.

**Extended Data Fig. 7:**
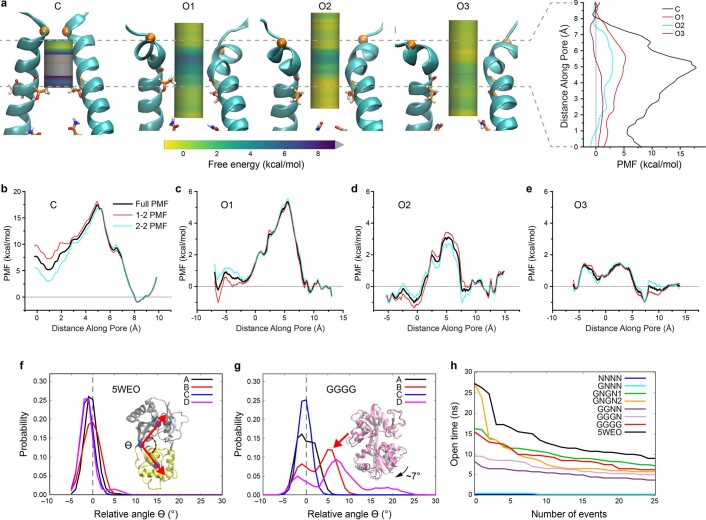
PMF in simulations of structures representing different conductance states and relationship between LBD clamshell closure and channel opening. **a**, Cartoons show the gate region of GluA2-γ2. Only M3 segments of subunits B and D are shown, with subunits A and C omitted for clarity. Cα positions for T625 residues are indicated by orange spheres, while T617 residues are shown in sticks. Bars in the middle of each structure are colored along the z-axis according to the value of PMF. For comparison, the plot on the right shows superposition of PMF curves for all structures. Ions progressing through the pore in PMF simulations of O3 and C are illustrated in Supplementary Videos 4 and 5, respectively. **b-e**, Estimates of uncertainty in PMF calculations computed using two halves of the stimulated data for C (b), O1 (c), O2 (d) and O3 (e) states. **f-g**, Distribution of the LBD clamshell closure angle in different subunits (A-D) during simulations of 5WEO (**f**) and GGGG (**g**) structures. The inset in (**f**) shows that the relative clamshell closure angle *θ* was calculated between the vectors (red) drawn from the hinge between D1 (grey) and D2 (yellow) to the D1 and D2 centers of mass (blue spheres). The *θ* value in 5WEO structure was set to 0, and all other *θ* angles were calculated relative to angles in 5WEO structure. The inset in (**g**) shows a D1-based superposition of LBDs from 5WEO (subunit A, grey) and GGGG (subunit B, pink) structures. **h**, Distribution of the open channel dwell time during simulations of different structures.

To classify the identified structural clusters according to pore opening and channel conductance, we first counted the water molecules travelling through the gate region of the pore in one direction as a measure of pore permittivity at zero membrane potential^47,48^ (Fig. 4b, Extended Data Figs. 5f, 6d and Supplementary Video 2). Substantially different water permittivity correlated well with the number of T617 side chains exposed to the pore (Extended Data Fig. 6c), and bending of M3 at A618 (Extended Data Fig. 6a, b) was responsible for the pore size as reflected by the T625 distances (Extended Data Fig. 6d). At the same time, similar water permittivity values were observed in the structures that contributed to the same cluster composed of structures from different trajectories (Fig. 4a, b). Second, we directly estimated ion conductance by performing non-equilibrium MD simulations of selected representative structures under an applied voltage (Extended Data Table 3). As ion channel permeation is rare on the MD time scale at physiological voltages and ion concentrations, it is common in simulations to use increased voltages (600 mV) and ion (K^+^) concentrations (300 mM)^46,52,53^. Such MD simulations provide a semiquantitative estimate of the channel conductance because of non-physiological settings and short durations that result in low-count statistics and high fluctuations in the number of permeating ions (Fig. 4c). Thus, we also performed umbrella sampling MD simulations to measure a permeant ion (K^+^) potential of mean force (PMF) (Fig. 4d and Extended Data Fig. 7a–e), which reflects a free-energy cost for an ion to leave an electrolyte solution and pass through the channel gate^54–58^. Substantially different ion conductance and PMF values calculated for the representative structures were highly consistent with water permittivity at equilibrium. A higher PMF barrier at the gate region of the channel indicated lower pore ion conductance. Combining all three approaches (water permittivity, ion conductance under applied potential and a single-ion PMF), the identified structural clusters were divided into four discrete groups that we propose to represent non-conducting C and conducting O1–O3 states (Fig. 5).

## Subconductance levels

The secondary structure of M3 that dictates the size of the gate opening and the T617 side chain orientation that controls both the extent of the pore constriction and its hydrophobicity appear to be signatures of different conductance states. Pore hydrophobicity increased when the T617 methyl group pointed towards the pore centre, whereas the pore became larger and more hydrophilic by hiding the methyl group into the channel wall and exposing the T617 hydroxyl group into the pore. In the non-conducting state C, represented by the NNNN and GNNN structures, the M3 segments formed straight α-helices, and the methyl groups of all four T617 pointed towards the pore centre ((i) in Fig. 5a). The lowest conductance state O1 is characterized by a relatively narrow pore, with two or three T617 residues that partially occlude the pore and one ((ii) in Fig. 5a) or two ((iii) in Fig. 5a) M3 helices (subunits B or D) bent at the gating hinge A618. Structures with two bent M3 helices closely resemble the GNGN1, GNGN2, GGNN, GGGN and GGGG structures. The O2 state is characterized by three or four T617 hydroxyl groups lining the pore, with two ((iv) in Fig. 5a) to four ((v) in Fig. 5a) bent M3 helices. Although O2 was populated for a significant fraction of time during simulations of several structures, including GNGN1, GNGN2 and GGGG, the 5WEO structure showed the closest resemblance to O2 with two bent M3 helices and all four T617 groups lining the pore ((iv) in Fig. 5a). One conformation, observed in simulations of the 5WEO and GNGN2 structures and characterized by M3 bending in three subunits, A or C in addition to B and D ((vi) in Fig. 5a), exhibited significantly higher conductance values and probably represents O3. Our MD simulations also revealed a conformation in which M3 segments of all four subunits are bent at the gating hinge A618 ((v) in Fig. 5a, and Supplementary Video 3). Although this conformation never reached higher than O2 conductance during simulations, we expect it can reach a wider separation of the M3 backbones and wider pore opening over longer simulations and represents a transient conformation that leads to O4.

To produce channel conductance, the LBDs appear to act in a strictly cooperative manner. For the channel to open to the first conductance level O1, both AD and CB LBD dimers should have at least one monomer bound to Glu. Although binding of one Glu molecule per LBD dimer creates a substantial separation of D2 lobes in the same dimer (d_635_ > 25 Å), channel opening requires separation of the D2 lobes in both AD and CB dimers (Extended Data Fig. 4c, d). In fact, an increased separation of the D2 lobes in the AD dimer of the GNNN structure is compensated by a reduced separation of the D2 lobes in the CB dimer, thus making the average separation of the D2 lobes in the GNNN structure comparable to the NNNN structure and smaller than in the conducting structures. Separation of the D2 lobes in individual LBD dimers increases the horizontal distance from the axis of the two-fold rotational symmetry (*L*) to D2 in diagonal subunits B and D, but not A and C of the receptor, and does not change the vertical position (*H*) of D2 relative to the channel (Extended Data Fig. 4e–g). The horizontal splaying of the D2 lobes between diagonal subunits B and D (average *L*) represents a driving force applied to the LBD–TMD linkers that leads to the opening of the AMPA receptor channel to the first and second conductance levels O1 and O2, respectively (Extended Data Fig. 4h–j). This also completes the link between the LBD clamshell closure and ion channel conductance and highlights the crucial role of subunits B and D in the opening of the channel to conductance levels O1 and O2, during which their M3 segments are kinked at the A618 gating hinge.

A comparison of dynamics of the fully liganded 5WEO and GGGG structures further sheds light on how a higher ligand concentration increases the propensity of higher conductance states. Simulations of the 5WEO structure showed that the LBD clamshell is tightly closed in all four subunits (Extended Data Fig. 7f), and the channel spends a longer time being continuously open (Extended Data Fig. 7h). By contrast, GGGG structure simulations revealed fluctuations and partial opening of two LBD clamshells (Extended Data Fig. 7g), shorter-living openings of the pore that result in the shorter total open-pore time (Extended Data Fig. 7h) and no conversions to higher conducting states (Supplementary Table 5). This dynamic behaviour explains why the 5WEO structure obtained at a high Glu concentration is associated with the O2 state, whereas the channel in the GGGG structure operates on par with partially liganded structures associated with the O1 state. Last, our molecular modelling of the 5WEO and GNGN2 structures suggests that opening of the channel to the third and fourth conductance levels O3 and O4, respectively, may involve kinking of the M3 segments in subunits A and C in addition to subunits B and D. Whether this implies a more crucial role of subunits A and C in channel opening to conductance levels O3 and O4 remains to be explored.

## Summary

We determined structures of the GluA2–γ2 complex in non-desensitizing conditions with various occupancy levels of the LBDs by Glu and showed that subunit cooperativity and low efficiency coupling of Glu binding to channel opening favour low levels of channel conductance. Our MD analysis suggests that the cryo-EM structural ensemble of the gate region (Fig. 3) represents only a narrow range of possible conformations, including representatives of C (NNNN and GNNN; (i) in Fig. 5a), O1 (GNGN1, GNGN2, GGNN, GGGN and GGGG; (iii) in Fig. 5a) and O2 (5WEO; (iv) in Fig. 5a) states. These three states are most abundantly observed in functional recordings (Fig. 1e). It is unclear, however, why other conformations predicted by electrophysiology or MD simulations, including alternative O1 ((ii) in Fig. 5a), O2 ((v) in Fig. 5a) and most importantly O3 and O4 conformations, have not been revealed by cryo-EM. One reason for such a deficiency is the inability of existing cryo-EM data analysis programs to resolve structures from small subsets of particles that represent rare conformations. More probable, however, the experimental cryo-EM conditions create a bias to populate only a certain subset of protein structures that represent longer-living local minima of this flexible protein energy landscape. Perhaps additional relative rearrangements of the fully closed ligand-bound LBD clamshells within the LBD layer, which were not favoured in our cryo-EM experiments, are required to facilitate transitions of the GluA2–γ2 complex to the O3 and O4 states. Therefore, to capture structures of other states of the dynamic structural ensemble, one would need to either change the structural experiment conditions, including iGluR and auxiliary subunit types, or use alternative tools, such as the MD simulations used in this work.

## Methods

### Construct for large-scale protein expression

The fusion construct GluA2–γ2 was prepared by introducing a GT linker between a modified rat GluA2_flip_ subunit with Q at the Q/R site (Q586), which was previously called GluA2* (ref. ^59^) and mouse γ2 C-terminally truncated after L207 (ref. ^60^). GluA2–γ2 was introduced into a BacMam vector for baculovirus-based protein expression in mammalian cells^61^, with the C-terminal thrombin cleavage site (LVPRG) followed by eGFP and an octa-His affinity tag (WSHPQFEK).

### Protein expression and purification

GluA2–γ2 bacmid and baculovirus were made using standard methods^61^. The P1 and P2 viruses were produced in Sf9 cells (Gibco, 12659017) and added to HEK-293S GnTI^–^ cells (American Type Culture Collection (ATCC), CRL-3022) incubated in FreeStyle 293 medium (Gibco, 12338018) at 37 °C and 5% CO_2_. Twelve hours after transduction, the cells were supplemented with 10 mM sodium butyrate and the temperature was changed to 30 °C. Seventy-two hours after transduction, the cells were collected by low-speed centrifugation (5,500*g*, 10 min), washed with 1× PBS (pH 8.0) and pelleted again (5,500*g*, 15 min). The cell pellet was resuspended in ice-cold lysis buffer, which contained 150 mM NaCl, 20 mM Tris pH 8.0, 1 mM β-mercaptoethanol (βME), 0.8 μM aprotinin, 2 μg ml^–1^ leupeptin, 2 μM pepstatin A and 1 mM phenylmethylsulfonyl fluoride (PMSF). Cells were subsequently lysed using a Misonix sonicator with a preset program (6 cycles of 15 s on at the amplitude of 8 followed by 15 s off; this program was repeated three times for optimal cell lysis) under constant stirring on ice. The lysate was centrifuged (9,900*g*, 15 min) to remove unbroken cells and cell debris, and the supernatant was subjected to ultracentrifugation (186,000*g*, 40 min) to pellet the cell membranes. The membrane pellet was mechanically homogenized and solubilized for 2 h at 4 °C in buffer that contained 150 mM NaCl, 20 mM Tris-HCl pH 8.0, 1 mM βME and 1% digitonin (Cayman Chemical Company, 14952). Insoluble material was removed by ultracentrifugation (186,000*g*, 40 min). The supernatant was added to Talon resin (Takara, 635504, 2 ml resin per 1 litre of the initial cell culture) and the mixture was rotated for 10–14 h at 4 °C. The protein-bound resin was washed with 25 ml of buffer that contained 150 mM NaCl, 20 mM Tris-HCl pH 8.0 and 0.05% digitonin, and the protein was eluted using the same buffer supplemented with 250 mM imidazole. To remove eGFP and the octa-His affinity tag, the eluted protein was subjected to thrombin digestion (1:200 w/w) for 1.5 h at 22 °C. The digest reaction was injected into a Superose 6 10/30 GL size-exclusion chromatography column (GE Healthcare) equilibrated with buffer that contained 150 mM NaCl, 20 mM Tris-HCl pH 8.0 and 0.05% digitonin. The tetrameric GluA2–γ2 peak fractions were pooled, concentrated to approximately 4 mg ml^–1^ and used for cryo-EM sample preparation. All the steps, unless otherwise noted, were performed at 4 °C.

### Cryo-EM sample preparation and data collection

To prepare the GluA2–γ2 samples, we used 300-mesh R1.2/1.3 commercial ultra Au foil Au/Au grids (EMS, Q350AR1.3A) or in-house Au/Au grids prepared as described in the literature^62^. In brief, the in-house grids were made by first coating C-flat (EMS) CF-1.2/1.3-2Au holey carbon grids with about 50 nm of gold using an Edwards Auto 306 evaporator. Subsequently, an Ar/O_2_ plasma treatment (6 min, 50 watts, 35.0 sccm Ar, 11.5 sccm O_2_) was used to remove the carbon with a Gatan Solarus (model 950) Advanced Plasma cleaning system. The grids were again plasma treated with the Gatan Solarus system (H_2_/O_2,_ 20 s, 10 watts, 6.4 sccm H_2_, 27.5 sccm O_2_) or glow discharged with a PELCO easyGlow cleaning system (Ted Pella, 30 s, 15 mA) immediately before sample application to make their surfaces hydrophilic. Purified protein was supplemented with 20 µM Glu and 100 µM CTZ (Tocris) and incubated for 30 min on ice. An FEI Vitrobot Mark IV (Thermo Fischer Scientific) was used to plunge-freeze the grids after application of 3 µl protein solution at 4 °C, 100% humidity, with a blot time of 5 s, a wait time of 15 s and a blot force of 5.

Images for frozen-hydrated particles of GluA2–γ2 were collected on a Titan Krios transmission electron microscope (Thermo Fisher Scientific) operating at 300 kV and equipped with a post-column GIF Quantum energy filter with the slit set to 20 eV and a Gatan K3 Summit direct electron detection camera (Gatan) using Leginon 3.5. Four datasets (4,137, 1,644, 7,104 and 4,851 micrographs, with a total of 17,736 micrographs) were collected in the counting mode, with an image pixel size of 0.83 Å and a defocus range of −1.0 to −2.5 µm. The total dose of about 58.5 e^−^Å^−2^ was attained by using a dose rate of about 16 e^−^pixel^−1^s^−1^ across 50 frames for 2.5 s total exposure time.

### Image processing

The initial processing was carried out using Relion 3.1 (ref. ^63^) (Extended Data Fig. 1). Frame alignment was done using MotionCor2 (ref. ^64^). Contrast transfer function (CTF) estimation was performed using Gctf 1.06 (ref. ^65^) on non-dose-weighted micrographs, whereas subsequent data processing was done on dose-weighted micrographs. Following CTF estimation, micrographs were manually inspected and those with outliers in defocus values, ice thickness and astigmatism, as well as micrographs with lower predicted CTF-correlated resolution, were excluded from the rest of the processing pipeline (individually assessed for each parameter relative to overall distribution; no set threshold). About 3,000 particles were manually picked to generate two-dimensional (2D) classes that were used as templates to autopick 3,484,799 particles. Picked particles were iteratively classified two-dimensionally and three-dimensionally to identify a subset of 455,635 particles that represented the best-looking classes. A cryo-EM map of GluA2–γ2 (Electron Microscopy Data Bank (EMDB): EMD-7959), low-pass-filtered to 40 Å, was used as an initial three-dimensional (3D) reference. After Bayesian polishing and CTF refinement, the particles were refined all together to produce an overall 3D reconstruction at 3.96 Å resolution. To eliminate heterogeneity created by the ATD layer moving relative to the rest of the protein and the micelle around the TMD, we performed particle subtraction with a mask whereby these regions were omitted. As a confirmation of the reduced particle heterogeneity, the overall refinement of subtracted particles yielded a 3D reconstruction with improved 3.74 Å resolution. To sort particles on the basis of LBD conformations, we subjected them to multiple rounds of 3D classification and refinement with a mask covering the LBD layer only and identified seven unique classes comprising the total of 358,818 particles. To avoid the possibility of the same particles contributing to different classes, we subjected all these particles to multireference 3D classification with the LBD layer mask. To confirm that each class represented a single conformation, the corresponding particles were subjected to variability analysis in cryoSPARC 2.14 (ref. ^66^). None of them showed detectable conformational heterogeneity. The resulting seven unique classes were 3D refined and post-processed without a mask to reveal the corresponding 3D reconstructions of the LBD–TMD region. For all classes, the refinement was initially done using C1 symmetry. For classes that had a two-fold symmetrical LBD layer, the initial C1-symmetry refinement did not reveal any asymmetry in the TMD. For these, refinement was repeated using C2 symmetry and this process produced reconstructions with better quality maps and higher resolutions.

### Model building and refinement

The models of LBD–TMD in seven unique conformations were built in Coot 0.9.2 (ref. ^67^) using cryo-EM density maps and the open-state structure of GluA2–γ2 (PDB ID: 5WEO) as guides. The models were tested for overfitting by shifting their coordinates by 0.5 Å (using shake) in Phenix 1.18 (ref. ^68^), refining each shaken model against a corresponding unfiltered half map, and generating densities from the resulting models in Chimera. The resulting models were real-space refined in Phenix 1.18 and visualized in Chimera^69^ or Pymol 2.4.0 (ref. ^70^).

### Patch-clamp recordings

DNA encoding GluA2–γ2 (described in the ‘Construct for large-scale protein expression’ section) was introduced into a pIRES plasmid for expression in eukaryotic cells that were engineered to produce GFP through a downstream internal ribosome entry site^59^. HEK-293T cells grown on glass coverslips in 35-mm dishes were transiently transfected with 1–5 μg of plasmid DNA using Lipofectamine 2000 reagent (Invitrogen). Recordings were made 24–96 h after transfection at room temperature. Currents from whole cells, typically held at a –60 mV potential, were recorded using an Axopatch 200B amplifier (Molecular Devices), filtered at 5 kHz, and digitized at 10 kHz using low-noise data acquisition system Digidata 1440A and pCLAMP 10.2 software (Molecular Devices). The external solution contained (in mM) 140 NaCl, 2.4 KCl, 4 CaCl_2_, 4 MgCl_2_, 10 HEPES pH 7.3 and 10 glucose; 7 mM NaCl was added to the extracellular activating solution, which contained 3 mM Glu to increase solution exchange speed rate. The internal solution contained (in mM) 150 CsF, 10 NaCl, 10 EGTA and 20 HEPES pH 7.3. Rapid solution exchange was achieved with a two-barrel theta glass pipette controlled by a piezoelectric translator. Typical 10–90% rise times were 200–300 µs, as measured from junction potentials at the open tip of the patch pipette after recordings. Data analysis was performed using Origin 9.1.0 software (OriginLab).

### Planar lipid-bilayer recordings

Planar lipid-bilayer measurements were performed as previously described^29^. In brief, planar lipid-bilayers were formed from a 30 mM solution of synthetic lipid mix of 1-palmitoyl-2-oleoyl-glycero-3-phosphocholine (POPC), 1-palmitoyl-2-oleoyl-glycero-3-phosphoethanolamine (POPE) and 1-palmitoyl-2-oleoyl-glycero-3-phosphoglycine (POPG) at a 3:1:1 ratio (Anatrace, P516:P416:P616) in *n*-decane (Sigma-Aldrich). The solution was used to paint a bilayer in an aperture of about 250 µm in diameter in a Meca chip (Nanion). Each cavity in the chip contains an individual integrated Ag/AgCl microelectrode. Bathing solutions consisted of 150 mM KCl, 0.02 mM MgCl_2_, 1 µM CaCl_2_ and 20 mM HEPES (pH 7.2). All reagents (Sigma-Aldrich) were ultrapure (>99%). Bilayer capacitances were in the range of 7–15 pF.

The purified protein (10 ng ml^–1^) was added to the bilayer-forming lipid mix (1 volume of protein to 1 volume of the lipid mix) and incubated for 30 min at 30 °C. After the bilayers had been formed by painting on a Meca chip (Nanion), they did not show any single-channel activity. Only after the incubated protein–lipid mix was added by painting were the unitary currents recorded using an Orbit mini device (Nanion). Data were low-pass filtered at 20 kHz and digitized at 1.22 kHz controlled by pCLAMP 10.2 software (Molecular Devices). Single-channel conductance events, all-points histograms and other parameters were identified and analysed with Clampfit 10.3 software (Molecular Devices). Independent of the presence of the auxiliary subunit and not affecting the conductance values, the channel open probability was significantly changing during and between different experiments, which reflected the commonly observed ‘modal’ behaviour of AMPA receptors^7,18,22,29^. For analysis of single-channel currents, we used the high open probability mode. In the high open probability mode, it was much easier to spot the recordings for which more than one channel was incorporated into the lipid bilayer, especially as all our recordings were made in the presence of 100 µM CTZ. Only recordings with no more than four conductance levels and no more than four peaks in the amplitude histograms, respectively, were subjected to single-channel analysis. All recordings with more than one channel incorporated into the lipid bilayer were discarded from the analysis. All experiments were performed at room temperature. Statistical analysis was performed using Origin 9.1.0 (OriginLab). Statistical significance was calculated using one-way ANOVA followed by Fisher’s least significant difference test. All data are presented as the mean ± s.e.m.

### MD simulations system set-up

The initial atomic models for the seven systems NNNN, GNNN, GNGN1, GNGN2, GGNN, GGGN and GGGG (Extended Data Table 2) were obtained from the cryo-EM structures reported in the current study, whereas the models for 5WEO were obtained from PDB (PDB ID: 5WEO). All protein model systems started with the N-terminal residue T394 of the LBD and ended with the C-terminal residue G820 of the TMD, which represented truncated versions of the full-length protein structures and defined as models of the AMPA receptor LBD–TMD. The M1–M2 intracellular loop between residues Y549 and S565, which is missing in all cryo-EM structures, was re-modelled using Modeler 10.1 software^71^. Each protein complex was built and assembled with POPC membrane using CHARMM-GUI 3.2 Membrane Builder^72,73^. All systems were solvated with TIP3P water and neutralized by adding Na^+^ and Cl^–^ ions to the bulk solution until the salt concentration was 150 mM. All initial membrane–protein complex model systems for MD equilibration simulations were built with the tleap program in AMBERTOOLS18 (ref. ^74^). FF99SB-ILDN force field^75^ parameters were used for the protein, Amber Lipid14 FF for POPC lipid and Li/Mertz FF parameters for ions^75,76^. Free glutamate (GLF) parameters were previously created^77^, and general AMBER force field (GAFF)^78^ was used for CTZ. Details for the eight fully solvated systems are given in Extended Data Table 2.

### Equilibrium MD simulations

Starting with 8 initial systems, 14 MD simulations (Extended Data Table 2) were performed following the identical protocol as described here. Energy minimization was performed while keeping restraints on the Cα atoms. Next, water and ions were equilibrated at constant volume MD simulations as the temperature was gradually increased from 0 to 300 K with the restraints of 40 kcal mol^–1^ Å^–1^ on all protein and lipid heavy atoms. This was followed by the equilibration MD simulations for 100 ns at 1 atm and 300 K with a time step of 2 fs using the pmemd.cuda program of the AMBER18 molecular dynamics package^74^. The restraints on the protein residues were gradually reduced to 0.5 kcal mol^–1^ Å^–1^. The MD production runs were performed without any restraints using AMBER18 for 320 ns for systems GGGN and GNNN, and 440 ns for systems GNGN1 and GNGN2, before extending production runs for these systems using the ANTON2 supercomputer^79^ for another 1,125–1,350 ns (Extended Data Table 2). In subsequent analyses, we only used 1,000 ns trajectory for GGNN and GGGG systems and 2,000 ns trajectories for two replicas of the 5WEO system (Extended Data Table 2). All AMBER18 production MD runs were performed with the integration time step of 2 fs, at 1 bar pressure and 300 K temperature. We used the Langevin thermostat with a damping coefficient of 1 ps^–1^ and a semi-isotropic pressure-scaling algorithm with a pressure relaxation time of 5 ps as implemented in AMBER18. All covalent bonds with hydrogen atoms were constrained using the SHAKE algorithm^80^. Long-range electrostatics were calculated using the particle-mesh Ewald method^81^, with non-bonded Lennard–Jones and Coulomb interaction cut-off radius value of 8 Å. All ANTON2 production simulations were performed at the constant temperature of 300 K using the Nose–Hoover thermostat, and the pressure was kept at 1 bar using the barostat MTK with the interval 480 and semi-isotropic pressure-scaling. The integration time step was 2.5 fs.

### Analysis of the equilibrium MD trajectories

All MD analyses and data extraction were performed using CPPTRAJ^82^ available with AMBERTOOLS18 (ref. ^74^) and VMD 1.9.3 (ref. ^83^) using snapshots extracted at every 250 ps from all trajectories. Data for two simulations of NNNN, GNGN1, GGGG and 5WEO systems were averaged out, unless otherwise specified. Representative snapshot figures and videos were created using VMD 1.9.3.

### Channel water density and water permeation

Channel water density maps (aligned with the origin at the centre of mass of T625) were calculated at –45 Å < *Z* < 10 Å (Extended Data Fig. 5f). For all systems, the average water permeation per nanosecond was calculated as counts of the total downward (–*Z*) water permeation divided by the total simulation time. Each successful count represents a downward (–*Z*) passing water with the entry point of *Z* = –6 Å and the exit point of Z = –16 Å, whereby all snapshots are aligned with the centre of mass at T625 origin. Rapid movements of water were better captured when counted at 10 ps time frames compared with 250 ps time frames. We report recalibrated water permeation for all systems as supported by a good correlation (*R*^2^ = 0.985) of permeation data obtained from trajectory snapshots saved at 10 ps versus 250 ps (Supplementary Table 6). Water permeation data were calculated from 40-ns blocks of AMBER production trajectories selected at every 200 ns from all systems.

### Analysis of MD trajectories with machine-learning methods

We used the unsupervised machine-learning *K*-means clustering method implemented through CPPTRAJ to characterize conformations of the channel gate region. *K*-means algorithm works by a two-step process called expectation and maximization. The expectation step assigns each data point to its nearest centroid, whereas the maximization step computes the mean of all the points for each cluster and sets the new centroid. Input data for clustering included backbone (atoms C, O, N, CA, CB) root-mean-square deviations of AMPA receptor M3-gate residues S614 to T625, and variables of ten numbers of clusters defining ten centroids were selected as an input parameter based on splits of structural variation in multiple runs (Extended Data Fig. 6a, b).

*t*-Distributed stochastic neighbour embedding (*t*-SNE) clustering is an unsupervised clustering technique for dimensionality reduction and high-dimensional data visualization. *t*-SNE aims to take high-dimensional data and reduce the dimensionality such that neighbouring points in high-dimensional space maintain their relative proximity in the low-dimensional representation. The result of this method is a low-dimensional representation of a high-dimensional space such that points within each cluster are similar to one another, and points in disparate clusters are dissimilar. This transformation between high to low dimensionality is a nonlinear transformation. Crucially, this method only groups clusters of similar points, and there is no meaningful interpretation of the size or location in the *xy* space or the relative position of pairs of clusters.

Taking snapshots from all 14 simulations at every 250 ps and combining them in a single dataset, we performed *t*-SNE clustering using a variety of dihedral angles and pairwise distances of the AMPA receptor gate region. We used a previously described^84^ CUDA accelerated implementation of *t*-SNE. To identify distinct clusters generated by *t*-SNE, we used a custom hierarchical clustering method in conjunction with the Scikit Learn^85^ mean-shift algorithm. Of the metrics tested, the χ_1_ dihedral angle (defined as the dihedral angles specified by the T617 atoms N, C_α_, C_β_ and O_γ_) of the T617 residue combined with the T625 C_α_ A monomer to C monomer distance, the T617 C_α_ A monomer to C monomer distance, and the C_α_ B monomer to D monomer distance (further referred to as pairwise distances) most clearly demonstrated the correspondence with observed water permeation. The *t*-SNE algorithm requires pairwise distances between all data points as an input, for example, Euclidean distance. Because we included angles in our high-dimensional vectors, we converted angular data into pairs of sine and cosine coordinates and designed scaling factors to mix distance and angle data with equal weights (see Supplementary Information for details).

### Unsupervised cluster identification

*t*-SNE dimensionality reduction only outputs an unlabelled set of points that may or may not contain individual clusters. To identify distinct clusters, *t*-SNE mapping was subjected to an unsupervised clustering method. First, the mean-shift algorithm was used to identify small groups of clusters within larger clusters. An intentionally small bandwidth was chosen to ensure that groups of points, which were clearly contained in two distinct *t*-SNE clusters, did not end up in the same mean-shift identified cluster. Next, we performed hierarchical clustering to merge small clusters into our final clustering. To do this, we iterated over all pairs of the mean-shift clusters. If the distance between any pair of the mean-shift cluster centroids was within a pre-specified cut-off distance, we iterated over all pairs of points between the two clusters. If the distance between any pair of points was smaller than another pre-specified cut-off distance, the two clusters were merged into one. The resulting clusters were alphabetically labelled (Fig. 4a and Extended Data Fig. 6d). To characterize each identified cluster, the mean and standard deviation of the relevant features were taken over all points in a particular cluster. For scalar values, these were computed normally, but for angular points, the angular mean and standard deviation were taken. Supplementary Table 2 shows the angular means and standard deviations for the four T617 χ_1_ dihedral angles in each cluster.

Representative structures for each cluster ((i)–(vi) in Fig. 5a) were obtained by finding a frame in each cluster for which the feature vector was closest in Euclidean distance (taking periodicity into account) to the mean feature vector of the cluster as a whole.

### Umbrella sampling MD simulations to compute the PMF for K^+^ ions

Umbrella sampling (US) MD simulations were used to compute K^+^ ion PMFs along the ion channel axes in GluA2–γ2 structures, identified as C, O1, O2 and O3 conductance states. The representative structures were chosen from the cluster q for C, cluster b for O1, cluster p for O2, and cluster w for O3 (Fig. 5). For each of the selected structures, an initial configuration of the simulated system was taken from the long equilibrium MD simulations (Extended Data Table 2). To initiate US MD simulations, a single water molecule positioned close to the centre of the pore at the channel extracellular entrance was converted into a K^+^ ion, and a second water molecule was chosen far into the intracellular side of the membrane to serve as a restraining potential anchor. A series of 10-ps simulations were then performed to generate initial equilibrated configurations of the ion along the channel axes. The simulation parameters were the same in all simulations. To ensure that the structure of the protein does not significantly change during the US MD simulations, the positions of Cα atoms for residues in the M3 helices were restrained (force constant, 20.0 kcal mol^–1^ Å^–2^). To constrain the K^+^ ion at progressive positions (0.4 Å increment) along the pore centre, a harmonic umbrella potential (force constant, 25.0 kcal mol^–1^ Å^–2^) was applied between the ion and the selected anchor water molecule, which was also restrained at the position with the force constant of 100.0 kcal mol^–1^ Å^–2^. A 6-ns equilibrium simulation was then performed for each K^+^ position along the channel axis, maintaining all the restraining potentials as described above. The distance between the anchor water and the ion was recorded every 100 fs. The weighted histogram analysis method (WHAM)^86^ was used to compute PMFs.

To estimate errors in PMF calculations, the Monte Carlo bootstrap method was applied as implemented in WHAM code^86^. In this work, samples consisted of observed deviations in position of an ion from a specified quadratic energy well. To obtain uncertainty estimates, observations were sampled with replacement to generate a new population. For populations derived from a time series, the autocorrelation time of the series needed to be taken into account. For this, we simply sampled fewer points dependent on the scale of the autocorrelation time. In a population of *N* samples with an autocorrelation time of *t* time steps, instead of sampling *N* points from the population with replacement, we sampled *N*/*t* points. In our exploration, we computed the autocorrelation times by finding the time delta it takes for the correlation between points to fall by e^−2^. In addition, PMF convergence was assessed by splitting each umbrella trajectory into two halves (1-2 and 2-2) and a new PMF computed from halves of the data. A PMF simulation is considered converged when the difference of PMF 1-2 – PMF 2-2 is within the tolerance for the precision of the specific problem.

### MD simulations of ion conductivity under applied voltage

To establish a correlation between ion conductivity and the structure of the channel gate in the O1, O2 and O3 states identified by machine-learning from the equilibrium simulations, we performed MD simulations under the influence of an electric field. Several initial systems were selected as representative structures of the O1, O2 and O3 states from the *t*-SNE clustering analysis of the equilibrium MD trajectories (Extended Data Table 3). In each such pre-equilibrated system, additional K^+^ and Cl^–^ ions were added to the bulk solution to represent 300 mM of KCl. Each of the initial systems (Extended Data Table 3) was energy minimized, whereas all Cα atoms were held under restraint. This was followed by the constant pressure, constant temperature equilibration MD simulation for 6 ns at 1 atm and 300 K, respectively. The restraints of 1 kcal mol^–1^ Å^–1^ were kept on the protein residues during NPT equilibration. The constant volume and temperature MD production runs without any restraints were performed for 360 ns at 300 K. MD simulation protocols, including the force field parameters, were kept similar to the equilibrium AMBER18 MD simulations described in the ‘Equilibrium MD simulations’ section. An external static electric field was applied normal to the membrane (along the *Z* direction) with the efz = 0.08 kcal mol^–1^ A^–1^ e^–1^, as implemented in AMBER18 to achieve a 600 mV applied voltage across the membrane. For all simulated systems, the average ion permeation per nanosecond was calculated as counts of the total ion permeation events in the *Z* direction divided by the total simulation time. Each successful count represents a passing of an ion through the boundary points defined at the M3 gate residues T625 at the entrance to the channel and an approximate centre of mass of the backbone atoms of the selectivity filter residues (_586_QQGCD_590_). Similar high voltage, high ion concentration computational electrophysiology approaches have been used in recent MD simulations of AMPA receptors and other channels to acquire statistically relevant ionic currents^46,52,53^. Only upward ion permeation (in the direction from the selectivity filter towards the gate) was observed in all simulations, as was also reported in simulations of AMPA receptors and K^+^-selective channels^87^, presumably as a consequence of a high applied voltage.

### Reporting summary

Further information on research design is available in the Nature Research Reporting Summary linked to this paper.

## Online content

Any methods, additional references, Nature Research reporting summaries, source data, extended data, supplementary information, acknowledgements, peer review information; details of author contributions and competing interests; and statements of data and code availability are available at 10.1038/s41586-022-04637-w.

### Supplementary information

Supplementary Information This file contains supplementary methods and Supplementary Tables 1–7. The supplementary methods include a description of the supplementary methodology for clustering analysis. Supplementary Tables 1–7 present information about the structure stability in MD simulations, correlation of water permeation analysis from trajectories with different record frequencies, and complete information about the clustering analysis results.

Reporting Summary

Supplementary Video 1 Na^+^ ion permeation through the AMPA receptor channel gate in the absence of an applied voltage in the GNGN2 equilibrium structure simulation. The gate region (indicated with the horizontal black lines) is defined between T625 and T617 (stick representation). Na^+^ ions in the pore are shown as blue, red or green spheres. Specifically, a Na^+^ ion permeating through the gate is shown in green, whereas a Na^+^ ion crossing through the channel selectivity filter formed by residues Q586 and Q587 (stick models) is shown in red. The protein is shown as a ribbon and water is shown as red and white ball-and-stick representations.

Supplementary Video 2 Permeation of water through the AMPA receptor channel gate in the absence of an applied voltage in the GNGN2 equilibrium structure simulation. The gate region (indicated with the horizontal black lines) is defined between T625 and T617 (stick representation). Selectivity filter residues Q586 and Q587 are also shown in stick representation. The protein is shown as a ribbon, and water is shown as red and white ball-and-stick representations. Permeating waters are shown in a space-filled representation.

Supplementary Video 3 Dynamics of the open channel of the AMPA receptor in the four-fold symmetrical conformation. Shown is a 280 ns fragment of equilibrium MD simulation of the 5WEO structure. All four M3 segments develop a kink starting at the A618 position. T617 residues are shown as stick models.

Supplementary Video 4 Umbrella sampling MD-generated pathway of the K^+^ ion permeation through the gate of the open O3 structure. The side chains of T625 (top) and T617 (bottom) are shown as orange sticks. Water molecules within a 5-Å-radius cylinder centred on the pore gate are displayed as sticks; the K^+^ ion is shown in blue and water molecules within 3 Å radius of it are shown in space-filled representation. Each snapshot of the video is the last frame of each umbrella sampling simulation.

Supplementary Video 5 Umbrella sampling MD-generated pathway of the K^+^ ion permeation through the gate of the closed C structure. The side chains of T625 (top) and T617 (bottom) are shown as orange sticks. Water molecules within a 5-Å-radius cylinder centred on the pore gate are displayed as sticks; the K^+^ ion is shown in blue and water molecules within 3 Å radius of it are shown in space-filled representation. Each snapshot is the last frame of each umbrella sampling simulation.

## Figures and Tables

**Extended Data Table 1:**
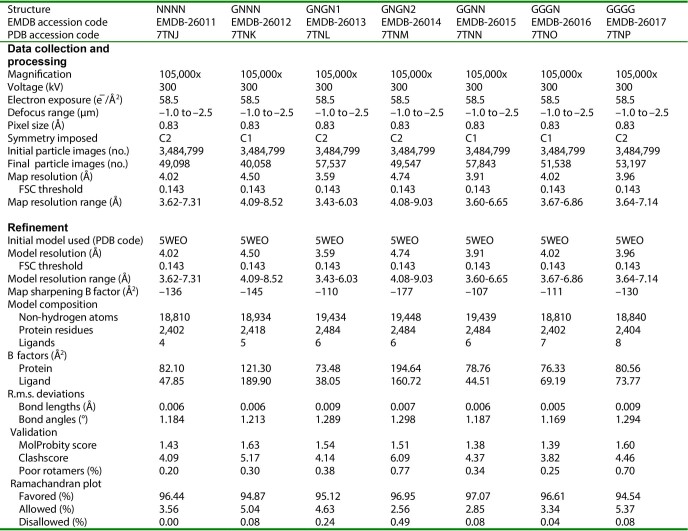
Cryo-EM data collection, refinement and validation statistics

**Extended Data Table 2:**
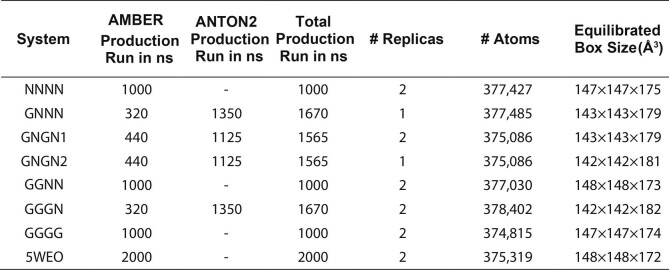
System information for the equilibrium MD simulations

**Extended Data Table 3:**
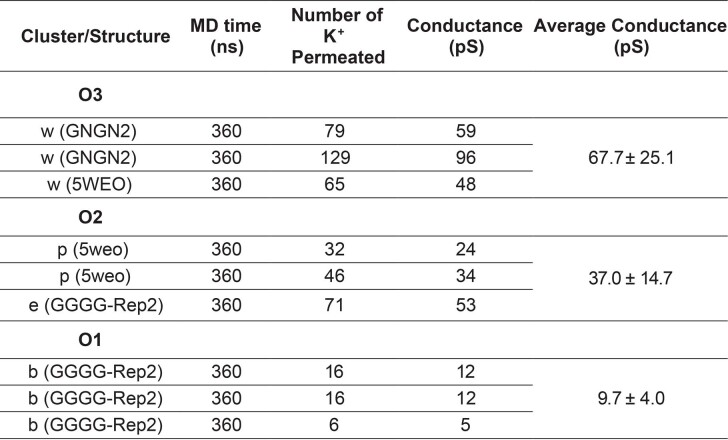
MD simulations of Ion currents under the applied voltage

## Data Availability

Cryo-EM density maps have been deposited to the EMDB under the accession codes EMD-26011 for NNNN, EMD-26012 for GNNN, EMD-26013 for GNGN1, EMD-26014 for GNGN2, EMD-26015 for GGNN, EMD-26016 for GGGN and EMD-26017 for GGGG (Extended Data Table 1). The corresponding model coordinates have been deposited to the PDB under accession codes 7TNJ for NNNN, 7TNK for GNNN, 7TNL for GNGN1, 7TNM for GNGN2, 7TNN for GGNN, 7TNO for GGGN and 7TNP for GGGG (Extended Data Table 1). All MD trajectories and raw data on PMFs, clustering and *t*-SNE analyses are available from the authors upon request.

## Code availability

Scripts for machine-learning analysis are available from the authors upon request.
