# Supplementary material for: Opening of glutamate receptor channel to subconductance levels
Source: Nature. 2022 Apr 20;605(7908):172–8. doi: 10.1038/s41586-022-04637-w (PMC9068512; doi:10.1038/s41586-022-04637-w)
Supplement: Supplementary file 1 — This file contains supplementary methods and Supplementary Tables 1–7. The supplementary methods include a description of the supplementary methodology for clustering analysis. Supplementary Tables 1–7 present information about the structure stability in MD simulations, correlation of water permeation analysis from trajectories with different record frequencies, and complete information about the clustering analysis results. [file 41586_2022_4637_MOESM1_ESM.pdf]

nature portfolio

https://doi.org/10.1038/s41586-022-04637-w

Supplementary information

Opening of glutamate receptor channel to subconductance levels

In the format provided by the authors and unedited

Supplementary Materials

Supplementary Methods

Data Preparation for TSNE Clustering

The first step in TSNE clustering is to compute the distance between all pairs of points. Traditionally, this is done by taking the Euclidean distance between all pairs of points. Because we include angles in our high-dimensional vectors, additional steps must be taken to ensure the validity of our pairwise distance matrix. In particular, we want to preserve the angular proximity of pairs of points which lie on either side of the periodic discontinuity (e.g., a pair of points with a particular angle that lies near $-\pi$ and π radians). To do this, we split every angular component of every high-dimensional input vector into sine and cosine components. This way, the points $-\pi$ and π radians with Euclidean distance 2π become the points (-1,0), (-1,0) with Euclidean distance 0 which accounts for the fact that these are the same point. This means that in order to account for periodicity, our 4-dimensional vectors representing the four T617 χ_1 dihedral angles are transformed to 8-dimensional vectors consisting of the sines and cosines of the original angles.

To mix the scalar data of the pairwise distances with the angular data of the T617 χ_1 dihedral angles, we note that for any angle, the maximum possible Euclidean distance is 2. As such we scale and shift all pairwise distances such that the largest observed distance has a scaled value of 2 and the smallest observed value has a scaled value of 0.

Characterizing Clusters

To characterize each identified TSNE cluster, the mean and standard deviation of relevant features were taken over all points in a particular cluster. For scalar values these were computed normally, but for angular points, the angular mean and standard deviation were taken. Table S1 shows the angular means and standard deviations of the four T617 χ_1 dihedral angles for each cluster. Note that in addition to the aforementioned 2 radian and 4 radian states, we also observe a 0 radian state. This radian state was observed infrequently and for the purposes of analysis was counted as a “non-obstructing” state.

Supplementary Tables

Supplementary Table 1: Stability of the ion channel gate in equilibrium MD simulations. The average values of the root-mean-square deviation (RMSD) from the initial structure calculated for the entire transmembrane domain (TMD) and the Gate region only (residues S615 to T625) for all simulated systems. The corresponding standard deviations are also listed. See Extended Data Table 2 for trajectory information.

	TMD RMSD		Gate RMSD	
Systems	RMSD (Å)	Std. Dev. (Å)	RMSD (Å)	Std. Dev. (Å)
NNNN	1.24	0.11	0.62	0.12
GNNN	1.31	0.10	0.68	0.07
GNGN1	1.74	0.17	2.20	0.93
GNGN2	1.47	0.14	1.31	0.24
NNGG	1.70	0.13	1.20	0.23
GNGG	1.41	0.12	1.25	0.18
GGGG	1.41	0.13	1.19	0.19
5WEO	1.78	0.16	1.84	0.28

Supplementary Table 2. 40 identified clusters from the TSNE analysis of the T617 sidechain χ_1 dihedral angles and pairwise distances. (**See Extended Data Fig. 6)**

Cluster	T617 Chi1 Mean				T617 Chi1 Std			
	A	B	C	D	A	B	C	D
a	4.074	4.125	4.102	2.147	0.146	0.149	0.143	0.160
b	2.211	2.232	4.087	4.116	0.171	0.220	0.137	0.137
c	4.092	4.048	2.218	2.251	0.139	0.162	0.169	0.156
d	4.084	4.074	4.091	2.242	0.137	0.148	0.133	0.152
e	4.094	2.243	4.084	4.106	0.128	0.156	0.139	0.147
f	2.242	4.107	4.097	4.113	0.155	0.130	0.184	0.121
g	2.220	4.086	4.089	2.224	0.308	0.138	0.133	0.175
h	4.060	4.117	2.204	4.100	0.147	0.127	0.157	0.137
i	4.052	2.236	2.251	2.244	0.152	0.295	0.236	0.150
j	4.062	4.111	4.086	4.125	0.151	0.139	0.144	0.150
k	2.243	2.261	4.044	2.252	0.157	0.146	0.137	0.141
l	4.069	4.118	4.084	4.113	0.144	0.132	0.169	0.130
m	2.272	4.056	2.234	2.222	0.164	0.151	0.166	0.260
n	2.231	4.047	2.218	4.112	0.151	0.179	0.180	0.154
o	2.247	4.084	4.082	4.124	0.149	0.157	0.132	0.143
p	4.085	4.082	4.090	4.094	0.133	0.151	0.133	0.149
q	2.241	2.241	2.240	2.224	0.151	0.151	0.154	0.157
s	4.099	2.262	4.092	2.186	0.124	0.185	0.127	0.202
t	2.294	4.055	4.049	2.199	0.161	0.145	0.126	0.153
u	4.095	2.263	2.262	4.113	0.134	0.153	0.152	0.128
v	4.041	4.107	4.040	0.213	0.149	0.130	0.546	0.161
w	4.093	4.135	4.090	4.113	0.107	0.133	0.137	0.136
x	2.234	2.186	2.264	2.237	0.151	0.139	0.157	0.144
y	2.204	2.242	2.248	4.102	0.186	0.223	0.181	0.131
z	4.060	4.047	4.049	4.066	0.135	0.142	0.138	0.140
aa	4.131	2.298	0.377	4.066	0.127	0.110	0.280	0.122
bb	2.231	4.071	2.173	2.240	0.176	0.144	0.174	0.145
cc	2.087	2.238	4.019	2.229	0.561	0.152	0.420	0.169
dd	4.085	4.064	2.233	4.105	0.131	0.179	0.161	0.145
ee	4.069	4.101	2.181	2.229	0.157	0.129	0.149	0.149
ff	4.029	0.401	2.300	4.080	0.135	0.187	0.132	0.171
gg	2.278	4.070	4.149	0.276	0.145	0.139	0.143	0.190
hh	2.243	4.112	2.225	4.100	0.159	0.124	0.153	0.131
ii	2.317	2.243	4.101	0.463	0.128	0.135	0.130	0.154
jj	4.065	0.345	4.127	4.071	0.127	0.154	0.145	0.263
kk	4.070	4.103	0.316	4.100	0.143	0.142	0.199	0.136
ll	2.240	4.090	4.106	4.108	0.151	0.134	0.146	0.128
mm	0.117	4.102	4.075	4.043	0.205	0.148	0.148	0.145
nn	2.158	4.134	4.108	4.129	0.182	0.146	0.126	0.160
oo	4.065	4.107	4.066	2.055	0.146	0.129	0.157	0.166

Supplementary Table 3. Cluster average and standard deviation values of the distances measured between the Cα atoms of T625 or T617 in diagonal subunits.

	T625 Mean Dist		T617 Mean Dist		T625 Std Dist		T617 Std Dist	
Cluster	AC	BD	AC	BD	AC	BD	AC	BD
a	28.509	24.920	13.234	12.560	0.874	0.897	0.444	0.459
b	14.849	26.345	12.141	13.196	2.330	2.396	0.674	0.722
c	12.077	22.203	11.868	12.131	1.735	8.344	0.575	0.548
d	14.712	26.529	12.184	13.304	2.583	4.285	0.990	0.821
e	14.315	26.690	12.479	13.398	2.540	2.126	0.809	0.644
f	22.885	29.101	11.479	13.577	1.169	0.853	0.480	0.722
g	18.123	27.546	11.670	13.164	2.618	1.431	1.002	0.800
h	20.103	29.045	11.908	14.105	2.525	1.256	0.600	0.919
i	10.486	12.598	11.233	11.717	1.259	5.906	0.501	0.474
j	29.077	26.400	13.672	13.573	1.202	2.548	0.605	0.844
k	10.596	11.240	11.026	11.619	1.155	4.149	0.479	0.441
l	22.807	29.675	11.590	13.987	1.454	1.225	0.498	0.757
m	10.741	13.135	11.575	11.594	1.216	6.814	0.470	0.536
n	12.311	27.173	12.000	12.754	1.798	5.996	0.474	0.808
o	15.271	26.757	12.683	13.274	2.340	3.023	0.565	0.853
p	15.173	27.908	12.803	13.579	2.651	1.577	0.835	0.913
q	10.275	10.298	11.098	11.382	0.651	1.042	0.462	0.450
s	13.089	25.760	12.040	12.903	1.802	3.222	0.716	0.544
t	10.713	15.067	11.498	11.898	0.744	8.883	0.435	0.509
u	10.605	25.069	11.606	13.065	1.216	3.140	0.518	0.599
v	21.706	28.461	11.367	13.122	2.438	1.033	0.514	0.596
w	24.658	30.995	13.965	14.756	1.129	1.167	0.606	0.668
x	15.741	27.595	10.365	11.029	0.652	0.583	0.389	0.593
y	10.262	22.863	11.493	12.564	1.174	6.785	0.440	0.747
z	10.415	16.052	11.881	11.950	1.103	8.136	0.331	0.343
aa	10.511	24.494	11.719	13.318	1.330	1.640	0.620	0.377
bb	17.105	28.040	10.978	13.388	1.518	0.887	0.603	0.643
cc	14.910	25.619	12.118	12.655	2.004	4.444	0.768	0.679
dd	12.669	26.779	12.149	12.904	1.626	5.082	0.610	0.845
ee	16.832	28.182	11.171	14.096	1.717	1.005	0.757	0.626
ff	11.362	27.381	11.750	13.053	1.630	1.957	0.523	0.683
gg	20.573	27.993	11.920	13.070	2.846	1.403	0.827	0.694
hh	18.739	28.349	11.433	14.335	1.598	1.021	0.614	0.767
ii	16.253	25.919	12.905	12.379	0.574	1.133	0.794	0.466
jj	15.525	26.831	12.784	13.427	1.638	0.857	0.673	0.697
kk	18.788	28.602	12.247	13.304	4.783	1.020	0.791	0.593
ll	18.386	28.432	11.505	14.124	1.680	1.424	0.481	0.937
mm	19.937	28.834	12.667	14.704	2.589	1.054	0.789	0.632
nn	28.039	27.124	13.301	13.886	1.287	0.953	0.589	0.703
oo	22.105	28.910	11.331	12.849	0.902	0.667	0.418	0.488

Supplementary Table 4. 40 identified clusters from the TSNE analysis of the T617 sidechain χ_1 dihedral angles and pairwise distances alongside the average number of water molecules that permeate the gate region immediately following the frame in the given cluster. Table is ordered according to the number of non-obstructing sidechains followed by the average permeation observed for the corresponding cluster. The last column shows the number of T617 sidechains in the non-obstructing configuration when counting the 0 radian state as a non-obstructing state.

	T617 Chi1 Mean				T625 Mean Dist		T617 Mean Dist		Avg Permittivity	No. "Obstructing"
Cluster	A	B	C	D	AC	BD	AC	BD	(water/ns)	Sidechains
q	2.241	2.241	2.240	2.224	10.275	10.298	11.098	11.382	0.056	4
x	2.234	2.186	2.264	2.237	15.741	27.595	10.365	11.029	0.220	4
k	2.243	2.261	4.044	2.252	10.596	11.240	11.026	11.619	0.441	3
i	4.052	2.236	2.251	2.244	10.486	12.598	11.233	11.717	1.137	3
m	2.272	4.056	2.234	2.222	10.741	13.135	11.575	11.594	1.207	3
y	2.204	2.242	2.248	4.102	10.262	22.863	11.493	12.564	2.761	3
bb	2.231	4.071	2.173	2.240	17.105	28.040	10.978	13.388	7.312	3
cc	2.087	2.238	4.019	2.229	14.910	25.619	12.118	12.655	8.485	3
t	2.294	4.055	4.049	2.199	10.713	15.067	11.498	11.898	1.287	2
u	4.095	2.263	2.262	4.113	10.605	25.069	11.606	13.065	4.240	2
c	4.092	4.048	2.218	2.251	12.077	22.203	11.868	12.131	5.553	2
n	2.231	4.047	2.218	4.112	12.311	27.173	12.000	12.754	7.184	2
b	2.211	2.232	4.087	4.116	14.849	26.345	12.141	13.196	8.352	2
ee	4.069	4.101	2.181	2.229	16.832	28.182	11.171	14.096	9.279	2
s	4.099	2.262	4.092	2.186	13.089	25.760	12.040	12.903	9.550	2
g	2.220	4.086	4.089	2.224	18.123	27.546	11.670	13.164	11.515	2
ii	2.317	2.243	4.101	0.463	16.253	25.919	12.905	12.379	12.156	2
hh	2.243	4.112	2.225	4.100	18.739	28.349	11.433	14.335	12.718	2
aa	4.131	2.298	0.377	4.066	10.511	24.494	11.719	13.318	3.729	1
a	4.074	4.125	4.102	2.147	28.509	24.920	13.234	12.560	6.878	1
dd	4.085	4.064	2.233	4.105	12.669	26.779	12.149	12.904	9.033	1
oo	4.065	4.107	4.066	2.055	22.105	28.910	11.331	12.849	9.712	1
gg	2.278	4.070	4.149	0.276	20.573	27.993	11.920	13.070	10.342	1
ff	4.029	0.401	2.300	4.080	11.362	27.381	11.750	13.053	10.424	1
e	4.094	2.243	4.084	4.106	14.315	26.690	12.479	13.398	11.103	1
d	4.084	4.074	4.091	2.242	14.712	26.529	12.184	13.304	11.149	1
o	2.247	4.084	4.082	4.124	15.271	26.757	12.683	13.274	11.742	1
f	2.242	4.107	4.097	4.113	22.885	29.101	11.479	13.577	12.395	1
ll	2.240	4.090	4.106	4.108	18.386	28.432	11.505	14.124	14.691	1
h	4.060	4.117	2.204	4.100	20.103	29.045	11.908	14.105	15.448	1
nn	2.158	4.134	4.108	4.129	28.039	27.124	13.301	13.886	19.227	1
z	4.060	4.047	4.049	4.066	10.415	16.052	11.881	11.950	3.096	0
v	4.041	4.107	4.040	0.213	21.706	28.461	11.367	13.122	11.219	0
j	4.062	4.111	4.086	4.125	29.077	26.400	13.672	13.573	13.477	0
jj	4.065	0.345	4.127	4.071	15.525	26.831	12.784	13.427	13.840	0
l	4.069	4.118	4.084	4.113	22.807	29.675	11.590	13.987	14.099	0
kk	4.070	4.103	0.316	4.100	18.788	28.602	12.247	13.304	14.428	0
mm	0.117	4.102	4.075	4.043	19.937	28.834	12.667	14.704	14.918	0
p	4.085	4.082	4.090	4.094	15.173	27.908	12.803	13.579	15.249	0
w	4.093	4.135	4.090	4.113	24.658	30.995	13.965	14.756	27.742	0

Supplementary Table 5. Makeup of simulations-based clusters derived from the TSNE analysis of the T617 sidechain χ_1 dihedral angles and pairwise distances. Entries are normalized over the number of points in each simulation, and each row sums to unity. The table is ordered by the number of points in each cluster.

Cluster	5WEO-Rep2	5WEO-Rep1	GNNN	GNGG-Rep1	GNGN2	GNGN1-Rep2	GNGN1-Rep1	NNNN-Rep1	NNNN-Rep2	NNGG	GGGG-Rep1	GGGG-Rep2	GNGG-Rep2	No. Points
p	0.023	0.081	0.000	0.208	0.113	0.008	0.107	0.000	0.000	0.148	0.088	0.191	0.033	19598
q	0.000	0.000	0.313	0.000	0.000	0.000	0.000	0.318	0.366	0.002	0.000	0.000	0.000	11688
l	0.694	0.287	0.000	0.000	0.001	0.013	0.002	0.000	0.000	0.003	0.000	0.000	0.000	5530
e	0.001	0.001	0.004	0.117	0.092	0.000	0.321	0.008	0.000	0.079	0.030	0.157	0.190	5413
j	0.003	0.002	0.000	0.000	0.000	0.994	0.000	0.000	0.000	0.000	0.000	0.000	0.000	4638
o	0.040	0.017	0.000	0.022	0.495	0.000	0.071	0.029	0.000	0.054	0.133	0.120	0.019	3079
u	0.000	0.006	0.030	0.040	0.001	0.000	0.012	0.009	0.005	0.000	0.032	0.001	0.864	2522
dd	0.003	0.005	0.050	0.074	0.001	0.000	0.130	0.018	0.000	0.090	0.538	0.000	0.093	2517
b	0.001	0.000	0.003	0.011	0.313	0.000	0.316	0.013	0.002	0.012	0.041	0.220	0.067	2373
h	0.534	0.376	0.000	0.001	0.029	0.015	0.007	0.000	0.000	0.011	0.027	0.000	0.000	2184
d	0.000	0.108	0.050	0.160	0.068	0.000	0.043	0.001	0.000	0.378	0.077	0.034	0.081	1871
ll	0.156	0.638	0.000	0.000	0.059	0.003	0.095	0.000	0.000	0.000	0.042	0.007	0.000	1451
y	0.000	0.000	0.054	0.009	0.022	0.000	0.000	0.177	0.048	0.033	0.006	0.000	0.652	1250
f	0.966	0.030	0.000	0.000	0.000	0.003	0.000	0.000	0.000	0.000	0.000	0.000	0.000	1131
a	0.001	0.000	0.000	0.000	0.000	0.999	0.000	0.000	0.000	0.000	0.000	0.000	0.000	1090
c	0.000	0.001	0.168	0.040	0.000	0.000	0.007	0.091	0.028	0.071	0.521	0.000	0.072	1026
s	0.000	0.000	0.017	0.052	0.064	0.000	0.087	0.020	0.005	0.170	0.011	0.051	0.522	813
ee	0.021	0.350	0.000	0.001	0.000	0.000	0.005	0.000	0.000	0.613	0.010	0.000	0.000	703
bb	0.000	0.642	0.000	0.000	0.000	0.000	0.000	0.000	0.000	0.297	0.060	0.000	0.000	651
g	0.124	0.436	0.000	0.000	0.183	0.003	0.106	0.000	0.000	0.092	0.056	0.000	0.000	647
i	0.000	0.006	0.344	0.016	0.048	0.000	0.000	0.192	0.363	0.000	0.005	0.000	0.027	572
m	0.000	0.000	0.208	0.000	0.000	0.000	0.000	0.407	0.211	0.043	0.132	0.000	0.000	562
n	0.031	0.006	0.028	0.022	0.000	0.000	0.000	0.067	0.000	0.119	0.629	0.000	0.099	494
z	0.000	0.000	0.430	0.045	0.000	0.002	0.002	0.220	0.000	0.018	0.177	0.021	0.085	388
k	0.000	0.023	0.307	0.000	0.002	0.000	0.000	0.279	0.370	0.000	0.000	0.000	0.019	383
oo	0.947	0.053	0.000	0.000	0.000	0.000	0.000	0.000	0.000	0.000	0.000	0.000	0.000	381
hh	0.121	0.873	0.000	0.000	0.000	0.000	0.000	0.000	0.000	0.000	0.006	0.000	0.000	334
cc	0.000	0.000	0.058	0.000	0.374	0.000	0.266	0.005	0.005	0.042	0.102	0.000	0.148	292
w	0.065	0.315	0.000	0.000	0.000	0.621	0.000	0.000	0.000	0.000	0.000	0.000	0.000	225
v	0.824	0.098	0.000	0.000	0.000	0.015	0.000	0.000	0.000	0.035	0.000	0.000	0.028	170
x	0.000	1.000	0.000	0.000	0.000	0.000	0.000	0.000	0.000	0.000	0.000	0.000	0.000	166
nn	0.015	0.015	0.000	0.000	0.000	0.970	0.000	0.000	0.000	0.000	0.000	0.000	0.000	159
t	0.000	0.000	0.263	0.000	0.006	0.000	0.000	0.439	0.000	0.009	0.284	0.000	0.000	137
gg	0.608	0.090	0.000	0.000	0.222	0.000	0.080	0.000	0.000	0.000	0.000	0.000	0.000	136
jj	0.000	0.000	0.000	0.228	0.142	0.000	0.567	0.000	0.000	0.000	0.000	0.063	0.000	49
kk	0.485	0.047	0.000	0.421	0.000	0.000	0.000	0.000	0.000	0.048	0.000	0.000	0.000	39
ii	0.000	0.000	0.000	0.000	1.000	0.000	0.000	0.000	0.000	0.000	0.000	0.000	0.000	30
aa	0.000	0.000	0.000	0.133	0.000	0.000	0.000	0.000	0.000	0.000	0.000	0.000	0.867	30
ff	0.000	0.000	0.000	0.112	0.439	0.000	0.000	0.000	0.000	0.000	0.000	0.000	0.449	26
mm	0.000	0.946	0.000	0.000	0.000	0.000	0.000	0.000	0.000	0.000	0.000	0.000	0.054	22

Supplementary Table 6: Correlation of water permeation throughout MD simulations. Shown is correlation of permeation values from the trajectory snapshots saved at 10 ps (Y) and 250 ps (X). Polynomial regression analysis shows correlation with $R^2 = 0.985$. See Methods section for calculation details.

	**From 250ps data**	**From 10ps data**	**For 10ps**	
	**X**	**Y**	**$y = 0.0757x^2 + 1.218x$**	
**Systems**	**Permeation per ns**	**Permeation per ns**	**Projected Values**	**Difference**
5WEO-Rep1	7.176	16.300	12.639	-3.661
5WEO-Rep1	5.752	8.900	9.511	0.611
5WEO-Rep1	6.248	10.325	10.565	0.240
5WEO-Rep1	6.952	12.225	12.126	-0.099
5WEO-Rep1	11.900	25.650	25.214	-0.436
5WEO-Rep1	11.324	25.675	23.500	-2.175
5WEO-Rep1	6.824	11.625	11.837	0.212
5WEO-Rep1	3.924	6.200	5.945	-0.255
5WEO-Rep1	5.652	9.300	9.302	0.002
5WEO-Rep1	7.300	13.150	12.925	-0.225
5WEO-Rep1	4.620	6.925	7.243	0.318
5WEO-Rep2	7.600	14.850	13.629	-1.221
5WEO-Rep2	7.124	10.275	12.519	2.244
5WEO-Rep2	6.400	12.150	10.896	-1.254
5WEO-Rep2	6.276	10.075	10.626	0.551
5WEO-Rep2	7.224	12.650	12.749	0.099
5WEO-Rep2	5.048	7.700	8.077	0.377
5WEO-Rep2	6.024	9.775	10.084	0.309
5WEO-Rep2	5.324	8.850	8.630	-0.220
5WEO-Rep2	10.224	20.800	20.366	-0.434
5WEO-Rep2	5.752	9.200	9.511	0.311
5WEO-Rep2	10.052	19.975	19.892	-0.083
GNGN1-Rep1	4.600	7.225	7.205	-0.020
GNGN1-Rep1	6.200	9.650	10.462	0.812
GNGN1-Rep1	6.876	12.900	11.954	-0.946
GNGN1-Rep2	4.576	7.050	7.159	0.109
GNGN1-Rep2	10.524	19.925	21.202	1.277
GNGN1-Rep2	5.924	9.725	9.872	0.147
GNGN2-Rep1	4.752	8.125	7.497	-0.628
GNGN2-Rep1	9.976	19.375	19.684	0.309
GNGN2-Rep1	11.848	24.500	25.057	0.557
NNGG-Rep1	6.800	11.675	11.783	0.108
NNGG-Rep1	6.276	10.275	10.626	0.351
NNGG-Rep1	5.876	9.725	9.771	0.046
NNGG-Rep1	8.024	13.625	14.647	1.022
NNGG-Rep1	2.648	3.500	3.756	0.256
NNGG-Rep1	7.524	12.225	13.450	1.225
GNGG-Rep1	5.224	8.725	8.429	-0.296
GNGG-Rep1	6.700	11.525	11.559	0.034
GNGG-Rep1	6.724	11.100	11.612	0.512

GGGG-Rep1	8.552	16.975	15.953	-1.022
GGGG-Rep1	3.700	5.875	5.543	-0.332
GGGG-Rep1	5.048	7.975	8.077	0.102
GGGG-Rep1	6.324	9.800	10.730	0.930
GGGG-Rep1	6.424	10.725	10.948	0.223
GGGG-Rep1	4.000	6.625	6.083	-0.542
GGGG-Rep2	6.276	9.925	10.626	0.701
GGGG-Rep2	8.552	16.050	15.953	-0.097
GGGG-Rep2	11.376	21.800	23.653	1.853
GGGG-Rep2	7.776	15.925	14.048	-1.877
GGGG-Rep2	5.024	8.000	8.030	0.030
GGGG-Rep2	5.076	8.075	8.133	0.058
GNNN	0.124	0.200	0.152	-0.048
GNNN	0.024	0.025	0.029	0.004
GNNN	0.000	0.050	0.000	-0.050
NNNN-Rep1	0.024	0.125	0.029	-0.096
NNNN-Rep1	0.052	0.075	0.064	-0.011
NNNN-Rep1	0.100	0.150	0.123	-0.027
NNNN-Rep1	0.100	0.100	0.123	0.023
NNNN-Rep1	0.176	0.300	0.217	-0.083
NNNN-Rep2	0.000	0.050	0.000	-0.050
NNNN-Rep2	0.100	0.100	0.123	0.023
NNNN-Rep2	0.024	0.025	0.029	0.004
NNNN-Rep2	0.024	0.075	0.029	-0.046
NNNN-Rep2	0.052	0.075	0.064	-0.011
NNNN-Rep2	0.000	0.000	0.000	0.000
			**R^2 = 0.985**	

Supplementary Table 7. Uncertainty Analysis of Ion permeation in non-equilibrium MD simulations with applied voltage (Extended Data Table 3). Each trajectory was divided into 40 ns non-overlapping blocks and conductance computed as described in Methods.

		**40 ns trajectory blocks**										
Simulation	**Cluster/ Structure**	**1**	**2**	**3**	**4**	**5**	**6**	**7**	**8**	**9**	**Avg. Conductance (pS)**	**Std. Error (pS)**
	**O3**											
**Sys1**	w(GNGN-2)	107.0	93.5	66.8	6.7	13.4	33.4	40.1	66.8	100.0	58.6	37.3
**Sys2**	w(GNGN-2)	160.0	107.0	140.0	60.1	0.0	60.1	100.0	120.0	113.0	95.6	48.5
**Sys3**	w(5WEO)	0.0	107.0	93.5	86.8	66.8	40.1	13.4	20.0	6.7	48.3	41.0
	**O2**											
**Sys1**	p(5WEO)	33.4	53.4	13.4	20.0	6.7	13.4	40.0	26.7	6.7	23.7	16.0
**Sys2**	p(5WEO)	0.0	26.7	107.0	93.5	33.4	6.7	6.7	6.7	26.7	34.2	39.3
**Sys3**	e(GGGG-Rep2)	40.1	80.1	40.1	60.1	20.1	33.4	73.4	66.8	60.1	52.7	20.1
	**O1**											
**Sys1**	b(GGGG-Rep2)	0.0	0.0	26.7	13.4	6.7	20.0	13.4	13.4	13.4	11.9	8.7
**Sys2**	b(GGGG-Rep2)	0.0	0.0	26.7	13.4	6.7	6.7	0.0	6.7	46.7	11.9	15.6
**Sys3**	b(GGGG-Rep2)	0.0	13.4	0.0	0.0	6.7	0.0	6.7	6.7	6.7	4.5	4.7
