# Supplementary material for: Opening of glutamate receptor channel to subconductance levels
Source: Nature. 2022 Apr 20;605(7908):172–8. doi: 10.1038/s41586-022-04637-w (PMC9068512; doi:10.1038/s41586-022-04637-w)
Supplement: Supplementary file 2 — Reporting Summary [file 41586_2022_4637_MOESM2_ESM.pdf]

nature portfolio

Corresponding author(s): Alexander Sobolevsky
Last updated by author(s): Feb 17, 2022

Reporting Summary

Nature Portfolio wishes to improve the reproducibility of the work that we publish. This form provides structure for consistency and transparency in reporting. For further information on Nature Portfolio policies, see our Editorial Policies and the Editorial Policy Checklist.

Statistics

For all statistical analyses, confirm that the following items are present in the figure legend, table legend, main text, or Methods section.

n/a	Confirmed	
☐	☒	The exact sample size (*n*) for each experimental group/condition, given as a discrete number and unit of measurement
☐	☒	A statement on whether measurements were taken from distinct samples or whether the same sample was measured repeatedly
☐	☒	The statistical test(s) used AND whether they are one- or two-sided<br>*Only common tests should be described solely by name; describe more complex techniques in the Methods section.*
☒	☐	A description of all covariates tested
☒	☐	A description of any assumptions or corrections, such as tests of normality and adjustment for multiple comparisons
☐	☒	A full description of the statistical parameters including central tendency (e.g. means) or other basic estimates (e.g. regression coefficient) AND variation (e.g. standard deviation) or associated estimates of uncertainty (e.g. confidence intervals)
☒	☐	For null hypothesis testing, the test statistic (e.g. *F*, *t*, *r*) with confidence intervals, effect sizes, degrees of freedom and *P* value noted<br>*Give P values as exact values whenever suitable.*
☒	☐	For Bayesian analysis, information on the choice of priors and Markov chain Monte Carlo settings
☒	☐	For hierarchical and complex designs, identification of the appropriate level for tests and full reporting of outcomes
☒	☐	Estimates of effect sizes (e.g. Cohen's *d*, Pearson's *r*), indicating how they were calculated

Our web collection on statistics for biologists contains articles on many of the points above.

Software and code

Policy information about availability of computer code

Data collection	Data on Titan Krios TEM equipped with a Gatan K3 Summit DED camera was collected using Leginon 3.5.
Data analysis	Origin 9.1.0, Leginon 3.5, MotionCor2, gCTF 1.06, RELION 3.1, cryoSPARC 2.14, UCSF Chimera 1.14, COOT 0.9.2, PHENIX 1.18, Pymol 2.4.0, Modeler 10.1, CHARMM-GUI 3.2, AMBER18, VMD 1.9.3, pCLAMP 10.2, Clampfit 10.3.

For manuscripts utilizing custom algorithms or software that are central to the research but not yet described in published literature, software must be made available to editors and reviewers. We strongly encourage code deposition in a community repository (e.g. GitHub). See the Nature Portfolio guidelines for submitting code & software for further information.

Data

Policy information about availability of data

All manuscripts must include a data availability statement. This statement should provide the following information, where applicable:

- Accession codes, unique identifiers, or web links for publicly available datasets
- A description of any restrictions on data availability
- For clinical datasets or third party data, please ensure that the statement adheres to our policy

Cryo-EM density maps have been deposited to the Electron Microscopy Data Bank (EMDB) under the accession codes EMD-26011 for NNNN, EMD-26012 for GNNN, EMD-26013 for GNGN1, EMD-26014 for GNGN2, EMD-26015 for GGNN, EMD-26016 for GGGN and EMD-26017 for GGGG (see Extended Data Table 1). The corresponding model coordinates have been deposited to the Protein Data Bank (PDB) under accession codes 7TNJ for NNNN, 7TNK for GNNN, 7TNL for GNGN1, 7TNM for GNGN2, 7TNN for GGNN, 7TNO for GGGN and 7TNP for GGGG (see Extended Data Table 1). All MD trajectories and raw data on PMF, clustering and TSNE analysis are available from the authors upon request.

Field-specific reporting

Please select the one below that is the best fit for your research. If you are not sure, read the appropriate sections before making your selection.

☒ Life sciences ☐ Behavioural & social sciences ☐ Ecological, evolutionary & environmental sciences

For a reference copy of the document with all sections, see nature.com/documents/nr-reporting-summary-flat.pdf

Life sciences study design

All studies must disclose on these points even when the disclosure is negative.

Sample size	No statistical methods were used to estimate an appropriate sample size.
Data exclusions	Poor 2D and 3D classes of particles were discarded during cryo-EM data processing.
Replication	For cryo-EM experiments, two independent maps of each reconstruction were generated in order to estimate resolution according to the 'gold standard' procedure. For electrophysiological experiments, all attempts at replication were successful.
Randomization	Randomization was not relevant to this study because no clinical trials or drug treatment assays were performed.
Blinding	Blinding was not relevant to this study because no clinical trials or drug treatment assays were performed.

Reporting for specific materials, systems and methods

We require information from authors about some types of materials, experimental systems and methods used in many studies. Here, indicate whether each material, system or method listed is relevant to your study. If you are not sure if a list item applies to your research, read the appropriate section before selecting a response.

Materials & experimental systems

n/a	Involved in the study	
☒	☐	Antibodies
☐	☒	Eukaryotic cell lines
☒	☐	Palaeontology and archaeology
☒	☐	Animals and other organisms
☒	☐	Human research participants
☒	☐	Clinical data
☒	☐	Dual use research of concern

Methods

n/a	Involved in the study	
☒	☐	ChIP-seq
☒	☐	Flow cytometry
☒	☐	MRI-based neuroimaging

Eukaryotic cell lines

Policy information about cell lines

Cell line source(s)	HEK293S GnTI-, ATCC, Cat#CRL-3022<br>HEK293T, ATCC<br>Sf9, Gibco, Cat#12659017
Authentication	None of the cell lines used have been authenticated
Mycoplasma contamination	The cell lines used have been tested for mycoplasma contamination by the providers (negative results) but have not been retested in the lab
Commonly misidentified lines<br>(See ICLAC register)	No commonly misidentified cell lines were used in this study
